# Morphology, Nucleation, and Isothermal Crystallization Kinetics of Poly(ε-caprolactone) Mixed with a Polycarbonate/MWCNTs Masterbatch

**DOI:** 10.3390/polym9120709

**Published:** 2017-12-13

**Authors:** Thandi P. Gumede, Adriaan S. Luyt, Mohammad K. Hassan, Ricardo A. Pérez-Camargo, Agnieszka Tercjak, Alejandro J. Müller

**Affiliations:** 1 Department of Chemistry, University of the Free State (Qwaqwa Campus), Private Bag X13, Phuthaditjhaba 9866, South Africa; tpgumede66@gmail.com; 2 Center for Advanced Materials, Qatar University, P.O. Box 2713 Doha, Qatar; mohamed.hassan@qu.edu.qa; 3 Polymat and Polymer Science and Technology Department, Faculty of Chemistry, University of the Basque Country UPV/EHU, Paseo Manuel de Lardizabal 3, 20018 Donostia-San Sebastián, Spain; riky0712@gmail.com; 4 Group ‘Materials + Technologies’ (GMT), Department of Chemical and Environmental Engineering, Faculty of Engineering, Gipuzkoa, University of the Basque Country UPV/EHU, 20018 Donostia-San Sebastián, Spain; agnieszka.tercjaks@ehu.es; 5 Ikerbasque, Basque Foundation for Science, 48013 Bilbao, Spain

**Keywords:** PCL, PC/MWCNTs masterbatch, nanocomposites, morphology, nucleation, conductivity, isothermal crystallization

## Abstract

In this study, nanocomposites were prepared by melt blending poly (ε-caprolactone) (PCL) with a (polycarbonate (PC)/multi-wall carbon nanotubes (MWCNTs)) masterbatch in a twin-screw extruder. The nanocomposites contained 0.5, 1.0, 2.0, and 4.0 wt % MWCNTs. Even though PCL and PC have been reported to be miscible, our DSC (Differential Scanning Calorimetry), SAXS (Small Angle X-ray Scattering), and WAXS (Wide Angle X-ray Scattering) results showed partial miscibility, where two phases were formed (PC-rich and PCL-rich phases). In the PC-rich phase, the small amount of PCL chains included within this phase plasticized the PC component and the PC-rich phase was therefore able to crystallize. In contrast, in the PCL-rich phase the amount of PC chains present generates changes in the glass transition temperature of the PCL phase that were much smaller than those predicted by the Fox equation. The presence of two phases was corroborated by SEM, TEM, and AFM observations where a fair number of MWCNTs diffused from the PC-rich phase to the PCL-rich phase, even though there were some MWCNTs agglomerates confined to PC-rich droplets. Standard DSC measurements demonstrated that the MWCNTs nucleation effects are saturated at a 1 wt % MWCNT concentration on the PCL-rich phase. This is consistent with the dielectric percolation threshold, which was found to be between 0.5 and 1 wt % MWCNTs. However, the nucleating efficiency was lower than literature reports for PCL/MWCNTs, due to limited phase mixing between the PC-rich and the PCL-rich phases. Isothermal crystallization experiments performed by DSC showed an increase in the overall crystallization kinetics of PCL with increases in MWCNTs as a result of their nucleating effect. Nevertheless, the crystallinity degree of the nanocomposite containing 4 wt % MWCNTs decreased by about 15% in comparison to neat PCL. This was attributed to the presence of the PC-rich phase, which was able to crystallize in view of the plasticization effect of the PCL component, since as the MWCNT content increases, the PC content in the blend also increases. The thermal conductivities (i.e., 4 wt % MWCNTs) were enhanced by 20% in comparison to the neat material. The nanocomposites prepared in this work could be employed in applications were electrical conductivity is required, as well as lightweight and tailored mechanical properties.

## 1. Introduction

Biodegradable polymers have received considerable attention due to their contribution to the reduction of environmental concerns and to the realization that global petroleum resources are finite. Different types of biodegradable polymers such as poly(ε-caprolactone) (PCL), poly(butylene succinate) (PBS), poly(lactic acid) (PLA), and poly(alkanoates) (PHA, PHB, PHBV) have been studied as potential biomaterials for a variety of applications such as biomedical devices, biodegradable packaging, adhesives, agricultural areas, auto-motion, and construction [1,2,3,4].

Amongst the biodegradable commercial polymers, PCL can be singled out due to its elasticity, biocompatibility, and good ductility caused by its low *T_g_* of −60 °C. It is also easy to be melt-processed by extrusion, melt-spinning, film blowing, and injection molding. However, it has relatively low mechanical strength, which limit practical applications. In order to enhance the thermal and mechanical properties of the matrix, and to produce thermally and electrically conductive materials, much research has been geared towards the production of conductive carbon-based bionanocomposites.

Conductive carbon-based fillers include carbon nanotubes (CNTs), carbon black (CB), graphite and carbon nanofibers (CNF). These materials have been extensively investigated due to their low density, inertness and good compatibility with most polymers [5]. However, CNTs have shown to have greater potential than any of the other carbon-based nanofillers for industrial applications, because of their unique one-dimensional structure with good electrical and thermal conductivity, and their excellent mechanical and thermal properties [6,7,8]. CNTs are extremely strong and stiff nanostructures of carbon atoms arranged in a cylindrical hexagonal network, and are often categorized in two different groups: single-walled carbon nanotubes (SWCNTs) and multi-walled carbon nanotubes (MWCNTs). SWCNTs consist of a single graphene layer rolled up into a seamless cylinder, whereas MWCNTs consist of two or more concentric cylindrical shells of graphene sheets coaxially arranged around a central hollow core with van der Waals forces between adjacent layers. MWCNTs are the ideal choice for high-volume industrial applications due to their bulk availability and better dispersion compared to SWCNTs [9,10,11,12].

Despite the advantages of carbon nanotubes, they have a tendency to form agglomerates during mixing with polymers. This is due to the van der Waals attraction between the nanotubes, which makes it difficult for them to be dispersed into polymers. Several methods have been used to enhance the dispersion of MWCNTs into polymer matrices [9,13,14,15]. The methods commonly employed to improve dispersion include (i) treatment of CNTs with inorganic solvents such as nitric acid (HNO_3_), sulfuric acid (H_2_SO_4_), and phosphoric acid (H_3_PO_4_), in order to attach hydroxyl and carboxyl acid functional groups to the nanotubes, and (ii) the masterbatch approach, which is a direct encapsulation of the MWCNTs into a polymer matrix, and the subsequent release of the carbon nanotubes into the matrix polymer during mixing in the melt. The masterbatch method has received great interest from an industrial point of view because it does not involve solvents that are harmful to the environment [13,14].

Nanocomposites of PCL with MWCNTs have potential applications in the biodegradable packaging market, biomedical field, and automotive industry, since the presence of the MWCNTs could overcome the limitations of PCL regarding thermal, mechanical, and electrical properties [16,17,18,19,20].

Several researchers investigated the nucleation and crystallization behavior of PCL/CNT nanocomposites with and without chemical modification [16,17,19]. Trujillo et al. [16] investigated the nucleation behavior of simple melt mixed, untreated PCL/MWCNT nanocomposites, and reported for the first time a supernucleation effect of approximately 200% in a well dispersed melt mixed system any chemical modification of the nanotubes (note that Trujillo et al. [16] obtained a very low dielectric percolation threshold of 0.3%). A similar or better supernucleation effect was also reported when PCL was blended with PCL-grafted MWCNTs (MWCNTs-*g*-PCL) [19]. In both studies, the supernucleation effect was attributed to the excellent dispersion of the MWCNTs in the polymer matrix, which was even better in the presence of functionalized MWCNTs. Pérez and co-workers [17] reported that MWCNTs, functionalized with 2-hydroxyethylbenzocyclobutene (BCB-EO) through a Diels–Alder cycloaddition reaction, nucleated linear PCL (L-PCL), but showed an antinucleation effect in cyclic PCL (C-PCL). This was due to a weak interaction between the MWCNT surfaces and the C-PCL because of the threading effect induced by the C-PCL molecules.

An evaluation of the mechanical performance of pristine and functionalized MWCNTs/PCL nanocomposites showed that functionalized MWCNT (*f*-MWCNT) nanocomposites gave much better mechanical properties than non-functionalized MWCNT nanocomposites. This was ascribed to a better dispersion of the functionalized MWCNTs in the PCL matrix as compared to non-functionalized MWCNTs. The better dispersion of the *f*-MWCNTs in the polymer matrix provided a more uniform stress distribution, minimizing the presence of stress-concentration centers, and increasing the interfacial area for stress transfer from the polymer matrix to the MWCNTs [12,18,21,22,23].

In this study, MWCNTs were dispersed into a PCL matrix through melt-mixing of the PCL with a PC/MWCNTs masterbatch. The structure and properties of the nanocomposites were correlated with the dispersion, morphology, and nucleating effect of the MWCNTs on the PCL matrix. Additionally, the efficiency of the nucleation and the overall crystallization kinetics of the PCL component were determined by self-nucleation and isothermal crystallization studies.

## 2. Experimental

### 2.1. Materials

A commercial PCL (CAPA 6500, Johannesburg, South Africa) was purchased from Southern Chemicals. It has a density of 1.1 g cm^−3^, a melting temperature of 58–60 °C, and a degree of crystallinity of ~35%. Its weight-average molecular weight (*M_w_*) and number-average molecular weight (*M_n_*) were measured by GPC, resulting in 113,400 g mol^−1^ and 73,620 g mol^−1^, respectively, with a polydispersity index (*M_w_*/*M_n_*) of 1.54.

A conductive masterbatch based on 85% low viscosity polycarbonate (Makrolon^®^ 2205 grade, *M_w_* of 20,100 g mol^−1^ [24]) loaded with 15 wt % MWCNTs (industrial grade NC7000) was obtained from Nanocyl (Sambreville, Belgium). It had a density of 1.175 g cm^−3^. The average diameter and length of the MWCNTs were, respectively, 10 nm and 3–4 µm. The carbon nanotubes contained more than 90% carbon and less than 10% metal oxide impurities.

The nanocomposites were prepared by melt-mixing in a twin-screw extruder (Thermo Scientific HAAKE Mini Lab II at the University of Pretoria, South Africa) operated under compressed air (100 rpm, 160 °C, 10 min). After extrusion, the samples were compression-molded at 160 °C for 5 min under 50 kPa using a hydraulic melt press. The calculated weight percentages of the different components in each of the investigated nanocomposites are given in Table 1.

### 2.2. Sample Characterization

Scanning electron microscopy (SEM) analyses were done in a JSM-7800F Extreme-Resolution Analytical Field Emission (JEOL, Tokyo, Japan) scanning electron microscope. The samples were mounted on aluminum pin stubs with steel epoxy glue and coated with gold to produce conductive coatings onto the samples.

For transmission electron microscopy (TEM) analysis, the samples were cryo-sectioned at −100 °C using a Leica UC-FC7 cryo-microtome. The 120-nm-thick sections were mounted on copper grids and viewed at room temperature using an FEI Tecnai 20 transmission electron microscope operating at 200 kV.

Atomic force microscopy (AFM) experiments were performed in selected samples at room temperature using a Bruker Multimode 8 scanning probe microscope equipped with a Nanoscope V controller. The micrographs, whose size was in a range of 0.6–5 μm, were obtained in tapping mode by using microfabricated silicon tips/cantilevers (cantilever spring constant, *k* = 42 N/m, and resonance frequency, *f*_0_ = 320 kHz (Bruker, Santa Barbara, CA, USA). Height and phase AFM images of lamellae and MWCNT were collected simultaneously and subjected to a first-order plane-fitting procedure to compensate the tilt. Both height and phase AFM images were similar, and consequently in this work only the phase AFM images will be reported. To obtain cross-section AFM images, samples were cut using an ultramicrotome Leica Ultracut R with a diamond blade.

Simultaneous SAXS/WAXS experiments were performed at the beamline BL11-NCD, ALBA Synchrotron facility in Barcelona, Spain. The samples were placed in DSC pans, and the DSC pans were put on a Linkam THMS600 hot stage coupled to a liquid nitrogen system. The hot stage was programmed to perform the crystallization and subsequent heating and at the same time register the SAXS/WAXS patterns. The thermal protocol was as follows: heating from room temperature to 100 °C, followed by holding for 3 min at 100 °C. Once the thermal history was erased, the samples were cooled down at 50 °C min^−1^ to the selected isothermal temperature. Different isothermal times were used depending on the temperature. Finally, after the isothermal step, the samples were heated at a rate of 5 °C min^−1^. The energy of the X-ray source was 12.4 keV (*λ* = 1.0 Å). In the SAXS configuration, the sample-detector (ADSC Q315r detector, Poway, CA, USA) with a resolution of 3070 × 3070 pixels, pixel size of 102 μm^2^ distance was 6495.0 mm with a tilt angle of 0°, whereas in the WAXS configuration, the sample-detector (Rayonix LX255-HS detector, Evanston, IL, USA) with resolution of 1920 × 5760 pixels, pixel size of 44 μm^2^ distance was 132.6 mm with a tilt angle of 21.2°. The intensity profile showed the plot of the scattering intensity as a function of the scattering vector, $q=4\pi sin\theta \lambda^{-1}$, where *λ* is the X-ray wavelength (*λ* = 1.0 Å) and *2θ* is the scattering vector. The scattering vector was calibrated using silver behenate (SAXS) and chromium (III) oxide (WAXS).

Dielectric relaxation measurements were performed using a Novocontrol GmbH Concept 40 broadband dielectric spectrometer (Montabaur, Germany), and data were collected over the frequency range 0.1–3 MHz at room temperature. Sample discs of 2 cm diameter were sandwiched between two gold-coated copper electrodes of 2 cm diameter and then transferred to the instrument for data collection. The AC conductivity was calculated, from the Novocontrol WinDETA software, by using the measured values of dielectric permittivity storage (ε′) and the dielectric loss factor (ε″).

Differential scanning calorimetry (DSC) analyses were performed under ultra high purity nitrogen gas flow in a power compensation Perkin Elmer Pyris-1 DSC, equipped with a refrigerated cooling system Intracooler 2P. The sample weight was ~5 mg in all cases.

For the non-isothermal DSC analyses, the samples were melted in the DSC for 3 min at 160 °C to erase any previous thermal history. The samples were then cooled at 20 °C min^−1^ from 160 to −20 °C, and then heated at the same rate from −20 to 160 °C.

The self-nucleation (SN) tests were performed according to a procedure established by Fillon et al. [25], and further developed and studied by Müller et al. [26,27,28]. The complete procedure is as follows:

(a) The sample was heated from 25 to 160 °C at 20 °C min^−1^ and maintained at that temperature for 3 min to erase thermal history.
(b) It was then cooled from 160 to −20 °C at 20 °C min^−1^ to create the initial “standard” state and held at that temperature for 3 min.
(c) It was then heated from −20 °C to a selected thermal treatment temperature or self-seeding temperature (*T_s_*), located in the final melting temperature range of the sample, and held at that temperature for 5 min.
(d) It was again cooled to −20 °C, where the effects of thermal treatment would be reflected in the crystallization behavior of the sample.
(e) Finally, it was heated to 160 °C, where the effects of thermal treatment would also be reflected in the melting behavior of the sample.

The most important parameters during SN are (1) the heating and cooling rates used, (2) the *T_s_* temperature, and (3) the time spent at *T_s_*.

The isothermal crystallization experiments were performed by following the procedure recommended by Lorenzo et al. [29] in which isothermal crystallization temperatures (*T_c_*) are chosen where no crystallization occurred during the cooling step from the melt (performed at 60 °C min^−1^). The samples were heated to 160 °C and kept at this temperature for 3 min to erase the thermal history. Then, a controlled cooling was applied, making sure that the cooling rate was 60 °C min^−1^, down to the set isothermal *T_c_* temperature. The sample was then kept at the set *T_c_* for a crystallization time (*t_c_*) until saturation was reached. Finally, the sample was heated from *T_c_* to 160 °C at 20 °C min^−1^ to record the melting behavior of the isothermally crystallized sample.

To determine the equilibrium melting temperatures,$\;T_{m}^{o}$, of the samples, the final step in the isothermal crystallization procedure, whereby the sample was heated at 20 °C min^−1^ in order to record the melting behavior of the isothermally crystallized polymer, was used to record the melting of the crystals formed at different crystallization temperatures, *T_c_*. The Hoffman–Weeks extrapolation [30] was then applied by plotting the observed melting temperature (*T_m(obs)_*) against *T_c_* to observe the intersection of this line with another line with a slope equal to 1 (*T_m_ = T_c_*).

Dynamic mechanical analyses (DMA) were performed from −100 °C to the onset of melting of PCL, which is ~50 °C, in the bending (dual cantilever) mode at a heating rate of 3 °C min^−1^ and a frequency of 1 Hz.

The tensile analysis of the samples was carried out using an Instron 4301 universal testing machine at a cross-head speed of 10 mm min^−1^. The dumbbell shaped samples had a Gauge length of 20 mm, a thickness of 1 mm, and a width of 5 mm. The samples were tested at a controlled ambient temperature of 23 °C and 50% relative humidity. Three samples of each composition were tested, and average values with standard deviations are presented.

Thermal conductivity measurements were performed using a Therm Test Inc. Hot Disk TPS 500 thermal constant analyzer. The instrument uses the transient plane source method. A 3.2 mm radius Kapton disk type sensor was selected for the analysis. The sample discs were 5 mm thick and 12 mm in diameter. The sensor was placed between two sample discs of the same composition. The measurements were made for a period of 25 s in order to prevent the heat flow from reaching the boundary of the samples. Three measurements were performed for each composition. The thermal conductivities are reported as average values with standard deviations.

## 3. Results and Discussion

### 3.1. Miscibility Assessment

The interaction between the components of a polymer blend can be determined from the composition dependence of the glass transition temperature (*T_g_*). If two polymers are completely miscible, only one *T_g_* is observed with its position determined by the composition of the blend. For immiscible polymer blends, two distinct *T_g_*-values are observed at the same temperatures as those of the parent homopolymers. However, when the two polymers are partially miscible, there are still two *T_g_*-values that will be shifted towards each other, with the degree of shift being dependent on both blend composition and miscibility degree [31].

**Figure 1:**
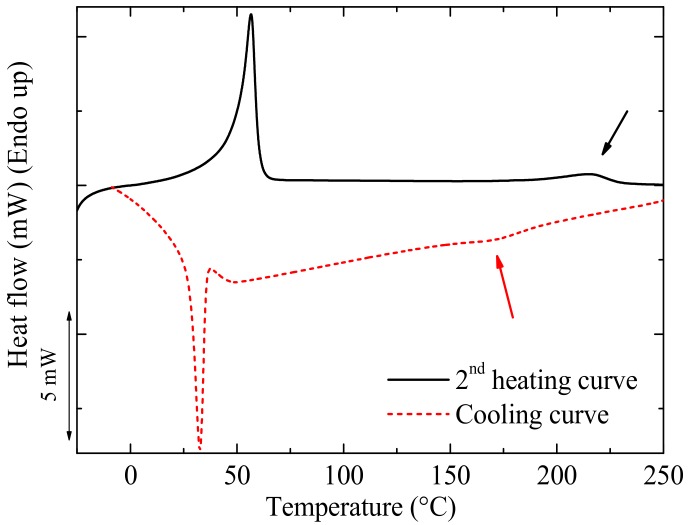
DSC (Differential Scanning Calorimetry) cooling and second heating curves for the selected 73/(23/4) *w*/*w* PCL/(PC/MWCNT) nanocomposite. The arrows indicate the crystallization and melting of the PC-rich phase in the blends.

In the present case, the *T_g_* of the PC component in the nanocomposites could not be observed through either DSC, DMA, or dielectric analysis (DEA) because PCL (the major component of the blends, i.e., the matrix) melted at a temperature well below the *T_g_* of PC (DMA analyses could not be performed at temperatures above the *T_m_* of PCL) and because PC crystallized in the nanocomposites (see crystallization and melting peaks indicated with arrows in Figure 1). PC does not normally crystallize, as it has a semi-rigid chemical structure and its crystallization is too slow. However, when plasticizers are added into the PC matrix, its free volume increases, which enhances the mobility of the PC polymer chains and its ability to crystallize can be enhanced [32,33,34]. In our case, the PCL obviously acted as a plasticizer for PC, a sign of miscibility (either full miscibility or partial miscibility). As PC crystallizes, its *T_g_* is difficult to observe by DSC, as the amount of mobile amorphous fraction per unit mass is very small in the blends.

**Figure 2:**
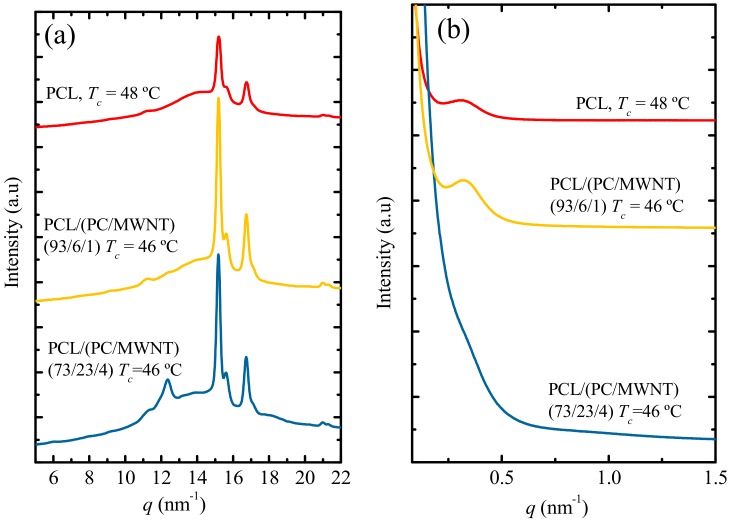
(**a**) WAXS (Wide Angle X-ray Scattering) diffractograms taken at selected isothermal temperatures; (**b**) SAXS (Small Angle X-ray Scattering) patterns taken at the same temperatures as in (**a**).

The crystallization of the PC component was also confirmed by simultaneous SAXS/WAXS analyses of PCL and the 93/(6/1) *w*/*w* PCL/(PC/MWCNTs) and 73/(23/4) *w*/*w* PCL/(PC/MWCNT) nanocomposite samples. Figure 2 depicts the final X-ray patterns taken under the indicated isothermal crystallization temperatures. The main WAXS reflections shown by neat PCL are also present in the nanocomposites, since the MWCNTs in the masterbatch acted only as nucleating agents (see Section 3.5). The main reflection peaks of PCL are located at *q*-values of 15.2 and 16.8 nm^−1^, and correspond to the (110) and (200) planes, respectively. It is worth noting that the characteristic shoulder in the PCL at 15.7 nm^−1^ appears in both neat PCL and the nanocomposites and corresponds to the (111) plane. All the reflections are consistent with the reported orthorhombic unit cell of PCL with unit cell parameters *a* = 7.48, *b* = 4.98, and *c* = 17.26 Å [35].

**Figure 3:**
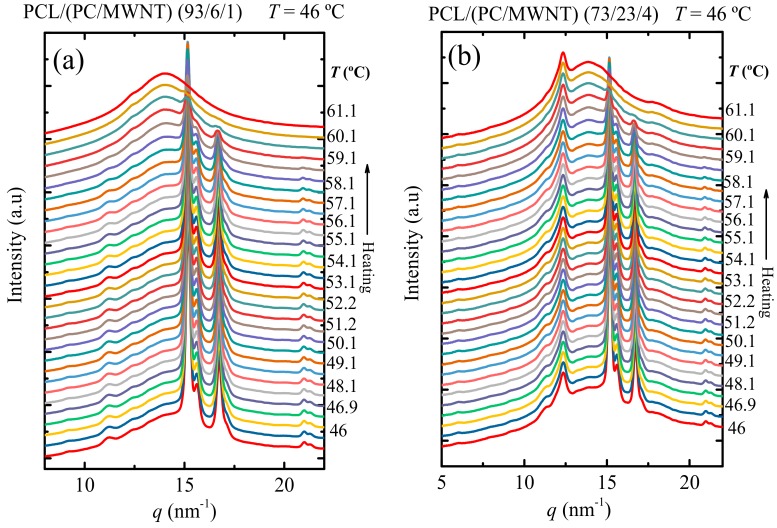
WAXS patterns taken during the heating at 5 °C min^−1^ after the isothermal step at 46 °C for (**a**) PCL/(PC/MWCNTs) (93/6/1) and (**b**) PCL/(PC/MWCNTs) (73/23/4).

In addition to the PCL unit cell peaks, there is a peak at 12.4 nm^−1^ (equivalent to a *2θ* of 17.4°), and this peak becomes pronounced as the PC content in the nanocomposites increases. This peak corresponds to the PC component that is able to crystallize due to the plasticization effect of the PCL, as will be shown in Figure 3. Figure 2b shows the SAXS patterns taken at the same condition used in the WAXS experiments. In these patterns, the PCL signal observed in the neat material and in the nanocomposites with low PC content is dominant, since the single peak corresponds mainly to the long spacing of PCL lamellae. However, at a higher PC concentration (i.e., 23 wt %), the PC is able to crystallize due to the plasticization effect of the PCL. Therefore, the SAXS signal is not clear due to the overlap of the long spacings generated by the lamellae of PCL and PC. The signal observed is probably an average of these two long spacings.

For the sake of clarity, WAXS patterns were taken during heating after the isothermal step (see Figure 3) for the selected samples of 93/(6/1) and 73/(23/4) *w*/*w* PCL/(PC/MWCNT) nanocomposites (the heating patterns of the other samples are shown in Figure S1). Figure 3 shows that the PC peak does not disappear when the PCL is already molten at *T* > 60 °C (see Figure 3). This behavior is clearly observed at higher PC concentrations in Figure 3b. According to our results and the literature [33,34], PC is able to crystallize, as mentioned earlier in the discussion, due to the plasticization effect of PCL and shows a main reflection at a 2*θ* angle of 17.1°. The peak at 12.4 nm^−1^ can therefore be attributed to the PC component, which crystallizes as a result of the plasticization effect of the PCL.

The *d*-spacings for all the reflections shown in Figure 2a were calculated according to Equation (1), whereas the long periods were calculated from the main PCL peaks in the SAXS patterns in Figure 2b. The relevant values are tabulated in Table 2.

(1) $$d^{*}=\frac{2\pi }{q_{max}}$$

The *d*-spacings and the d*-values of neat PCL and the PCL in the nanocomposites are almost the same for neat PCL and low PC concentrations (i.e., 6 wt %). In the case of 73/(23/4) *w*/*w* PCL/(PC/MWCNT) nanocomposite, the peak related to the PC component is the same as the one reported in the literature (0.464 nm) [33]. In the SAXS patterns, an overlap between the long spacings of PC and PCL occurs, which explains the decrease in d*-values in comparison with the other samples.

**Figure 4:**
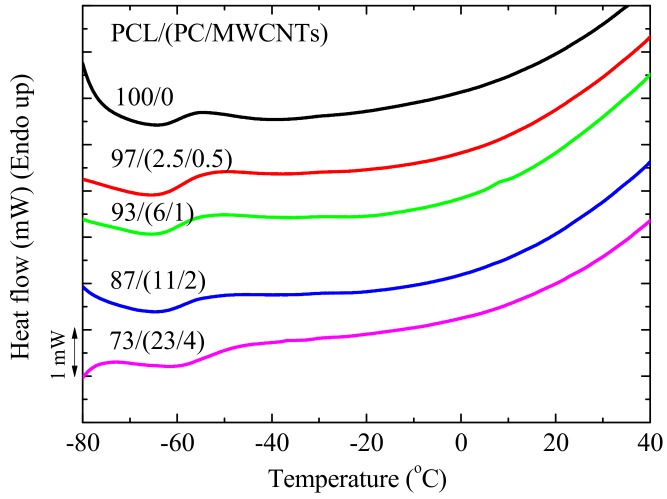
DSC heating curves for neat PCL and the PCL/(PC/MWCNT) nanocomposites, showing the glass transitions around −60 °C.

**Figure 5:**
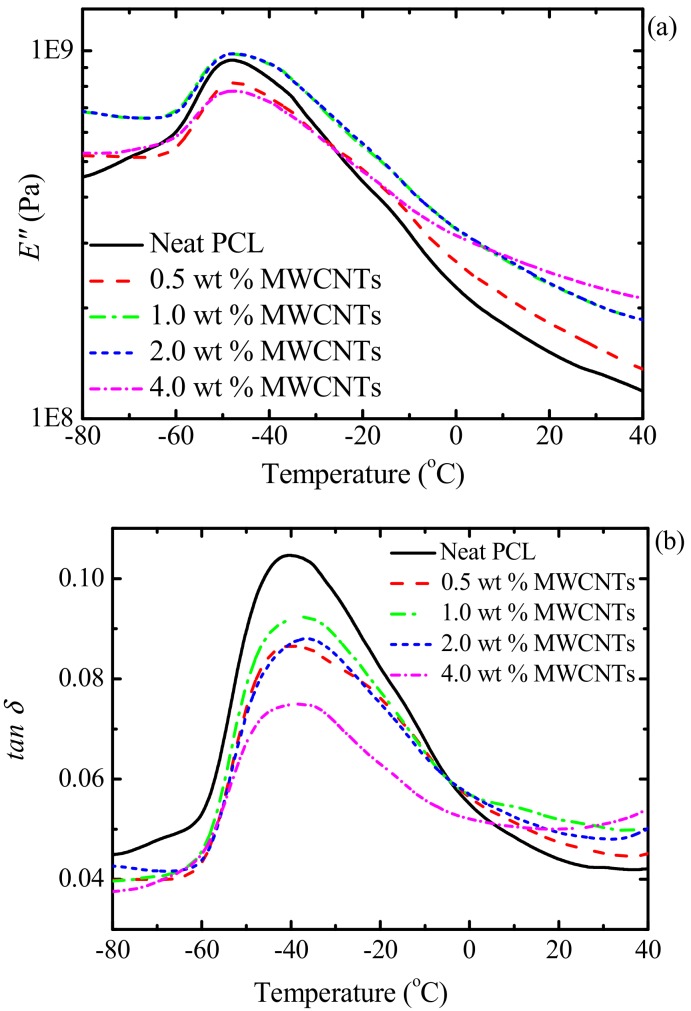
DMA (**a**) loss modulus (*E″*) and (**b**) tan δ curves for the investigated samples.

**Figure 6:**
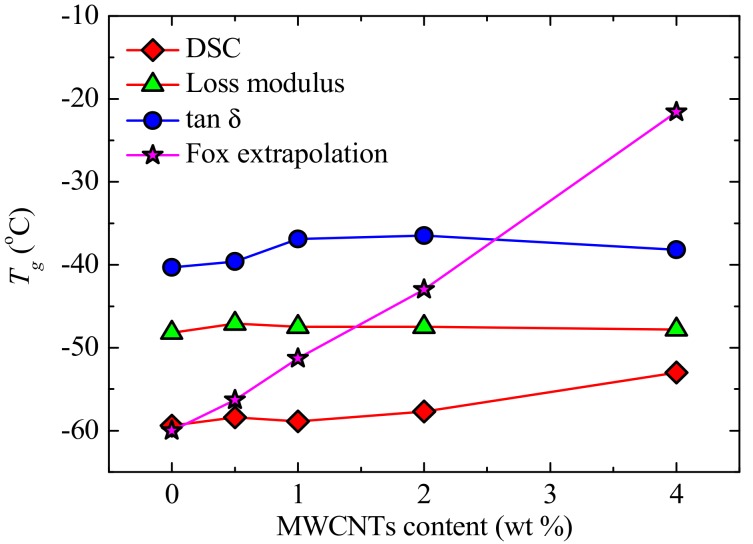
Glass transition temperatures of neat PCL and the PCL/(PC/MWCNT) nanocomposites as a function of MWCNT content.

The DSC and DMA results in Figure 4 and Figure 5 show little change between the *T_g_*-values for neat PCL and PCL within the different nanocomposites. The *T_g_*-values from the two techniques are different for the same sample, with *T_g,DSC_ < T_g,E_*_”_ *< T_g,tanδ_* (Figure 6). This is well-known, as DMA applies not only a heating rate but also a mechanical deformation with a particular frequency, which, as a result, increases the rate at which *T_g_* is being measured [36,37,38]. The trends from the different sets of results are, however, the same. The results show that the presence of the masterbatch had little effect on the *T_g_* of PCL, which may be an indication of limited interfacial interaction between the PCL and the PC in the masterbatch. Theoretically, when two polymers are completely miscible, the *T_g_* of the PCL in the blend nanocomposites should have increased to approximate values calculated according to the Fox equation (Equation (2)).

(2) $$\frac{1}{T_{g}}=\frac{w_{1}}{T_{g1}}+\frac{w_{2}}{T_{g2}}$$

where *T_g_* is the PCL/PC blend glass transition temperature, and *T_gi_* and *w_i_* are the respective glass transitions and weight fractions of PCL and PC. The glass transition temperatures of PCL obtained from both DSC and DMA did not change much across the composition range, and the values are lower than those predicted by the Fox equation (Figure 6), which could be an indication of immiscibility or partial miscibility between the PCL and PC.

However, a closer inspection of the DSC results (Figure 4) shows that the blend containing the highest amount of PC, i.e., 73/(23/4) PCL/(PC/MWCNTs) has a *T_g_*-value of approximately 7 °C higher than neat PCL (see also Figure 5). DMA results also show an increase in *T_g_*-values of this blend with respect to neat PCL (i.e., 3–4 °C). In fact, Figure 5 shows an increasing trend (much smaller than that predicted by the Fox equation but still significant) of *T_g_* with increases in PC content in the blend.

If complete immiscibility would be present in the blends, no plasticization of PC would have been observed (as indicated by PC crystallization, demonstrated above). Taking into account the results presented so far, we can conclude that the blends are partially miscible. Two phases are formed: (1) a PC-rich phase, where a small amount of PCL chains are present and can plasticize the PC component, such that it can crystallize, and (2) a PCL-rich phase, where the amount of PC chains present is very small, such that changes in the *T_g_* of the PCL phase are much smaller than those predicted by the Fox equation.

### 3.2. Electron Microscopy (SEM and TEM) and Atomic Force Microscopy (AFM)

SEM and TEM images for the PCL/(PC/MWCNT) nanocomposites with different PC/MWCNT contents were obtained to confirm the presence of two phases (PCL-rich and PC-rich phases), and to see whether any of the MWCNTs diffused into the PCL phase.

**Figure 7:**
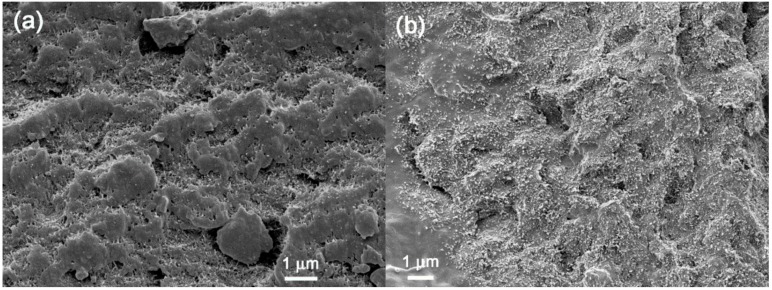

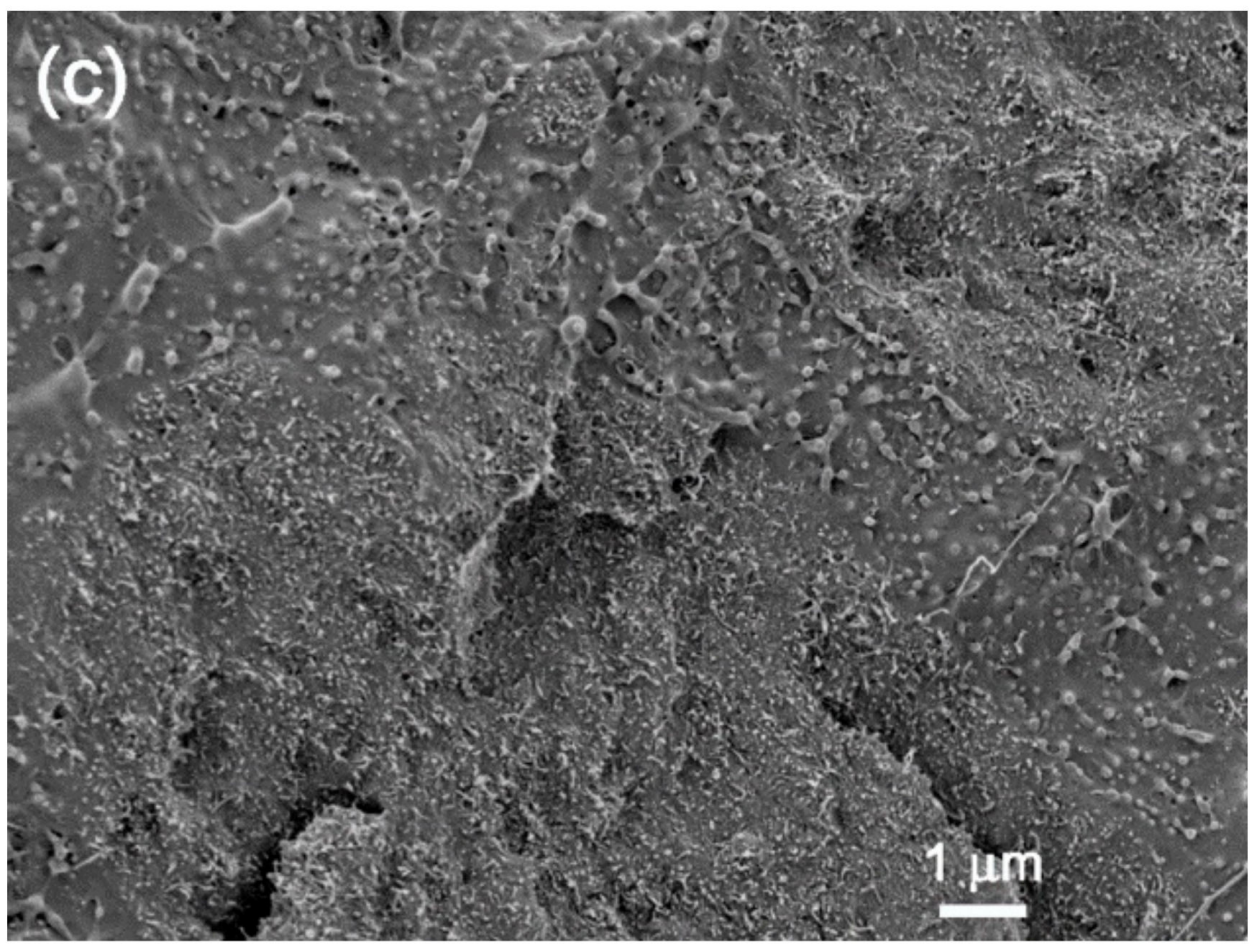
SEM micrographs for the PCL/(PC/MWCNT) nanocomposites, respectively, containing (**a**) 1.0 wt %, (**b**) 2.0 wt %, and (**c**) 4.0 wt % MWCNTs.

**Figure 8:**
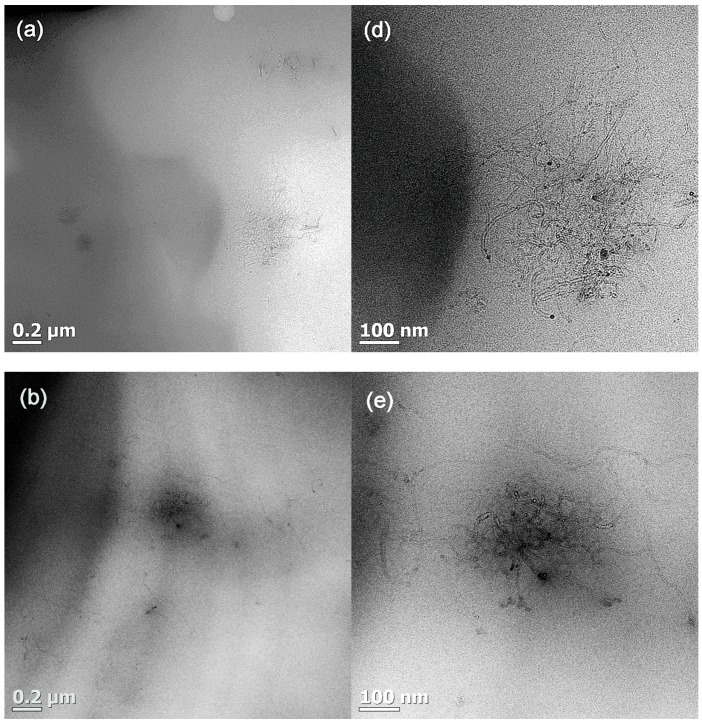

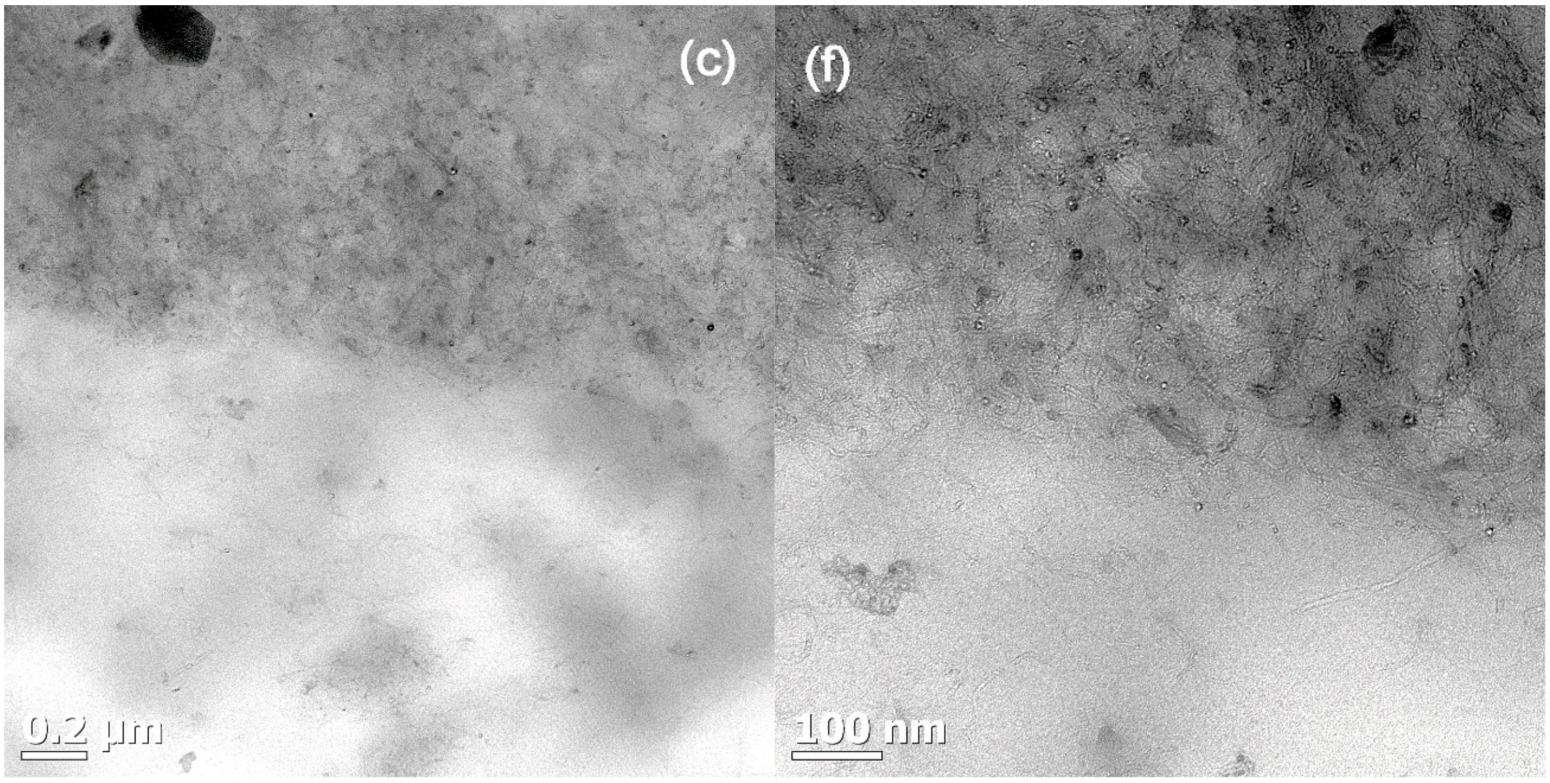
High and low magnification TEM micrographs for (**a**,**d**) 1.0 wt %, (**b**,**e**) 2.0 wt %, and (**c**,**f**) 4.0 wt % MWCNTs in the PCL/(PC/MWCNT) nanocomposites.

The SEM and TEM images in Figure 7 and Figure 8 show that there are no clear phase boundaries separating the PCL-rich and PC-rich phases, and that the MWCNTs were fairly well dispersed throughout the blend matrix, although there were areas where the MWCNTs were more concentrated that correspond to the PC-rich phase. The results corroborate the partial miscibility of the blends. If the blends were immiscible, all MWCNTs would be confined to the PC phase (as a PC-based masterbatch was employed). However, it is clear that, due to partial miscibility and the establishment of PC-rich and PCL-rich phases, a fair number of MWCNTs diffused from the PC-rich phase to the PCL-rich phase, even though areas were found where MWCNTs agglomerates were still confined to the PC-rich pockets.

Mixed reports about the miscibility of PC and PCL exists in the literature [20,34,39,40,41,42,43,44]. In our case, only partial miscibility was developed between the components.

Factors that determine whether the polymers are completely miscible or not include (i) molar-mass distribution, (ii) chemical structure, and (iii) molecular architecture of the components present [45,46]. The molecular weight of PC (20,100 g mol^−1^) in the masterbatch was appreciably lower than that of PCL (113,400 g mol^−1^). The PC crystallized in the presence of the PCL, which acted as a plasticizer and imparted enough mobility to the PC chains [32,33,34,43].

Shih et al. [47] studied the effect of molecular weight on the compatibility between blends of polycarbonate and poly(hexamethylene sebacate). They reported that, in all the blends prepared, the PC crystallized as a result of the plasticizing effect of the poly(hexamethylene sebacate). When a low molecular weight PC and a high molecular weight poly(hexamethylene sebacate) were used, the compatibility was enhanced because of the increased entropic contribution to the Gibbs free energy of mixing.

In our case, the PC crystallization probably reduced the miscibility between PC and PCL, making them only partially miscible. Another reason why we did not observe complete miscibility in our system, is that the MWCNTs probably had a strong interaction with the PC and with each other, which restricted the PC flow during the mixing process and resulted in a fair amount of the PC chains being unable to diffuse into the PCL-rich phase.

**Figure 9:**
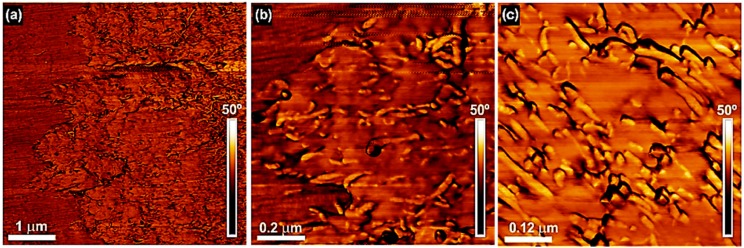
(**a**) Low and (**b**,**c**) high magnification AFM phase images for the 73/(23/4) *w*/*w* PCL/(PC/MWCNT) nanocomposite.

Figure 9 shows the atomic force microscopy (AFM) phase images for the 4 wt % MWCNT nanocomposite at different magnifications. It can be seen at low magnification (Figure 9a) that an interphase exists between the PCL matrix and the PC/MWCNT-rich phase. The interphase is clear but reveals a very intimate contact between the phases, which probably stems from partial miscibility. At higher magnification (Figure 9b), the MWCNTs can clearly be seen in the PC-rich phase (the one with the higher amount of nanotubes). However, in addition, some nanotubes can be observed crossing the interface from the PC-rich phase to the PCL-rich phase (see Figure 9b). Figure 9c shows that the MWCNTs are well dispersed within the PC-rich phase.

### 3.3. Dielectric Measurements

**Figure 10:**
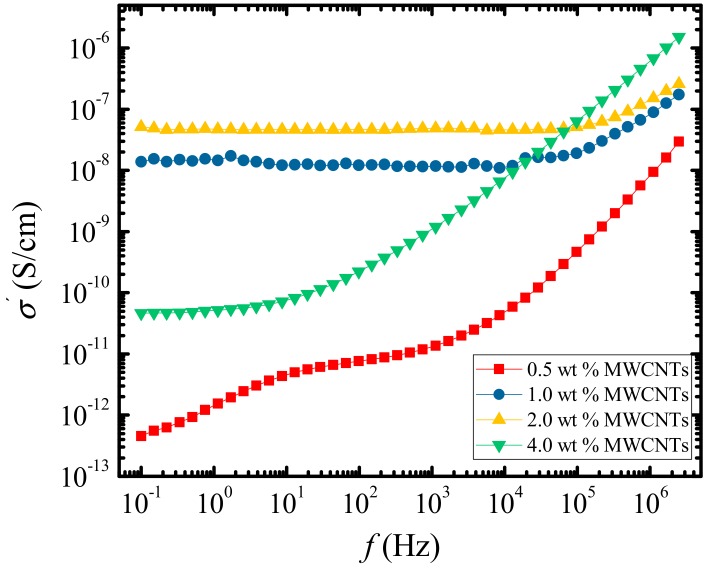
Conductivity vs. frequency at room temperature for the sample containing a different wt % of MWCNTs.

AC conductivity vs. frequency (*f*) plots at 20 °C for all samples are demonstrated in Figure 10. For the sample with 0.5 wt % MWCNTs, the trend does not show any plateau regions, and the conductivity is gradually increasing with increasing frequency, which is primarily due to dipolar motions of the PCL chains [48,49,50]. At such a low concentration of MWCNTs, the nanotubes did not achieve a percolated network structure.

The DC conductivity increases and the plateau widens to include most of the measured frequency range for samples with an MWCNT concentration of 1.0 and 2.0 wt %. There could be two reasons for this kind of conductivity behavior: Firstly, increasing MWCNT content may have resulted in an increased charge carrier concentration and therefore better interparticle contact and an easier formation of a continuous pathway for electron hopping. Secondly, sharp differences in dielectric constant and conductivity of the MWCNTs in comparison to PCL or PC surrounding matrices may lead to the Maxwell–Wagner–Sillars (MWS) interfacial polarization effects, as mobile charges get accumulated at the interface between the MWCNTs and the two matrices [50,51]. Other important parameters including MWCNT dipole density and mobility, as well as the mobility of the surrounding polymer chains, are necessary for the MWS polarization phenomena to occur.

As the MWCNT content is increased to 4 wt %, the conductivity dropped sharply, almost by two orders of magnitude, which could reflect aggregation of the nanotubes within the PCL matrix, as well as the crystallization of the PC phase. This aggregation could cause an accumulation of the mobile charges at the interfaces between the nanotubes and therefore act as barrier for long-range hopping events of the electrons, i.e., inter-nanotubes interfaces act as dead ends for the mobile charges [49].

From the obtained results the dielectric percolation threshold occurs between 0.5 and 1.0 wt % of MWCNTs or the critical concentration needed to form a connected conducting pathway for the electrons to hop between the nanotubes and throughout the surrounding polymer matrices. The formation of a percolation pathway also demonstrates that the MWCNTs were able to diffuse from the PC-rich phases to the PCL matrix. However, they did this more efficiently at lower PC concentrations. When the PC content was 23%, i.e., when the MWCNT content was increased to 4%, then aggregation of the nanotubes or less transfer of the nanotubes to the PCL matrix must have occurred. In fact, the AFM images shown in Figure 9 for the nanocomposite with 4% MWCNTs do show a much higher concentration of nanotubes in the PC-rich phase than in the PCL-rich phase.

**Figure 11:**
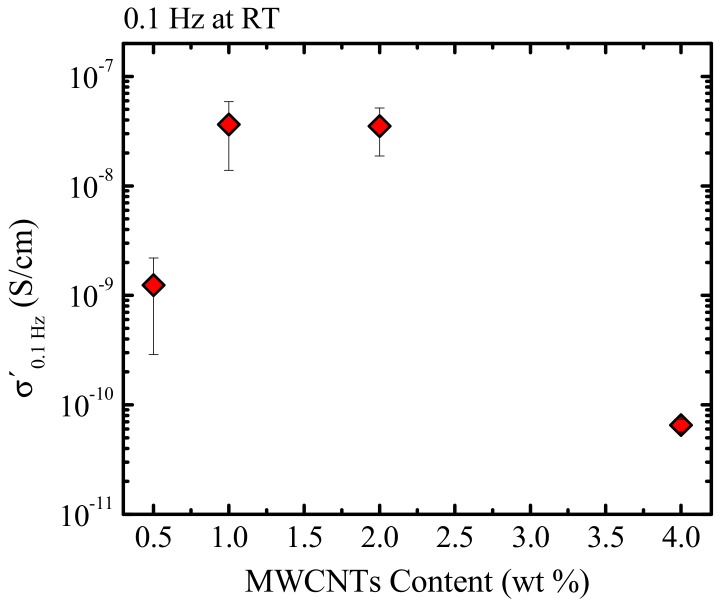
Conductivity vs. MWCNT content at room temperature and frequency of 0.1 Hz.

Figure 11 depicts the conductivity as a function of the MWCNT content at room temperature and at the lowest measured frequency (0.1 Hz). The plot reveals a sharp increase in the conductivity as the MWCNTs’ wt % increases from 0.5 to 1.0%, then a sharp drop after 2.0%. Trujillo et al. [16] reported a very low dielectric percolation threshold of 0.3 wt %, due to the excellent dispersion obtained in the PCL/MWCNT nanocomposites. Vega et al. [19] obtained a percolation threshold of 0.240 wt % for the same nanocomposites and 0.236 wt % for nanohybrids (PCL/MWCNT-*g*-PCL) determined by rheological measurements. It is worth noting that, in these systems [16,19] with such a low percolation threshold, a supernucleation effect (a nucleation efficiency of 200%) was reached. Higher percolation thresholds have been reported by Saeed et al. [52]. The authors obtained a percolation threshold of 2 wt %, for PCL/MWCNT nanocomposites prepared via in situ polymerization of the PCL on nitric acid-treated CNTs surfaces using rheological measurements. However, Pötschke et al. [53] reported values of 1 wt % of percolation thresholds for PC/MWCNT nanocomposites produced by melt-mixing using the masterbatch dilution method without any modification to the nanotubes. However, it is important to note the differences in the processing conditions used during the preparation of these nanocomposites, as the viscosity of the matrix greatly affects the dispersion of the nanotubes [49].

### 3.4. Non-Isothermal DSC

**Figure 12:**
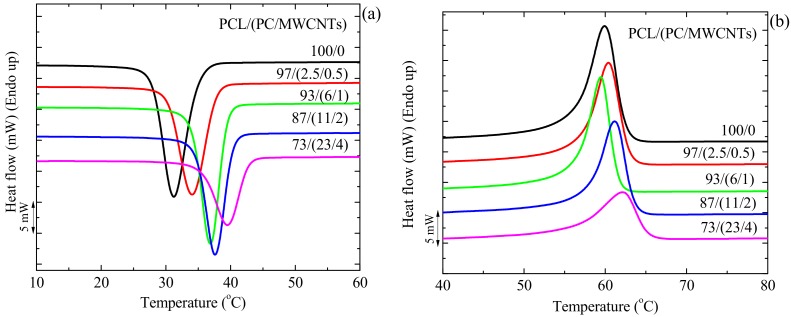
DSC (**a**) cooling and (**b**) second heating curves at 20 °C min^−1^ of neat PCL and the PCL/(PC/MWCNT) nanocomposites.

Figure 12 shows the DSC (a) cooling scans after erasing the thermal history and (b) the subsequent heating scans performed at 20 °C min^−1^ for the different investigated samples. The crystallization peak temperatures (*T_c_*) of PCL in the nanocomposites shifted to higher temperatures as compared to that of neat PCL (Figure 12a). The DSC heating curves show little or no change in the melting temperature (*T_m_*) of PCL in the nanocomposites compared to that of neat PCL (Figure 12b).

**Figure 13:**
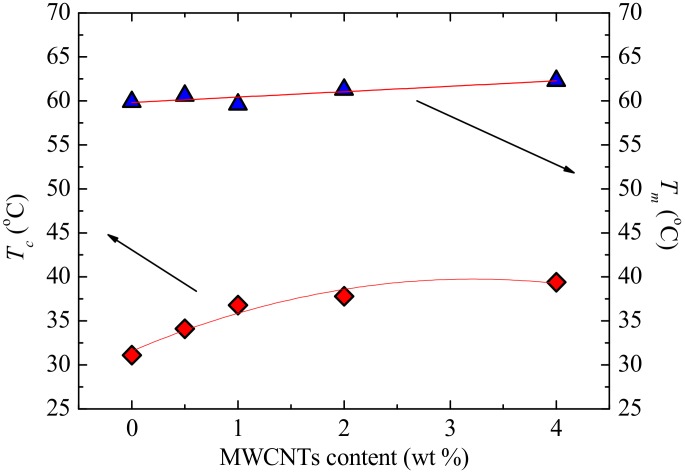
DSC crystallization and second heating melting temperatures as a function of MWCNT content for neat PCL and the PCL/(PC/MWCNT) nanocomposites. A linear fit and a polynomial fit for the experimental data of *T_m_* and *T_c_*, respectively, are used to guide the eye.

To examine the results presented in Figure 12, the *T_c_*- and *T_m_*-values were plotted in Figure 13 as a function of MWCNT content. The increase in *T_c_* with increasing MWCNT content clearly indicates a nucleation effect of the MWCNTs that penetrated into the PCL-rich phase (as was demonstrated morphologically by SEM and AFM images). However, a saturation of this nucleation effect starts below 2 wt % MWCNTs, in line with the percolation threshold of 0.5–1 wt % found in the previous section. This saturation is related to the aggregation of MWCNTs and the limited diffusion of the MWCNTs with increasing PC content. The *T_m_* remains almost constant with the increase in MWCNT content, as is expected when a nucleating agent is used. This is due to the metastable nature of polymer crystals that usually require large increases in *T_c_*-values to give rise to *T_m_*-values [16]. The nucleation action of the MWCNTs is further studied in the next section through self-nucleating experiments.

### 3.5. Self-Nucleation (SN)

**Figure 14:**
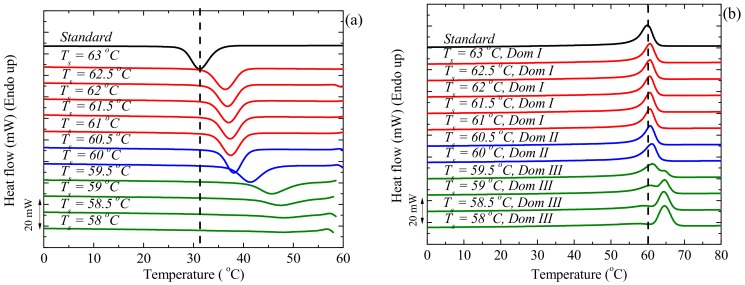
(**a**) DSC cooling scans for neat PCL after 5 min at the indicated *T_s_*, and (**b**) subsequent heating scans after the cooling runs shown in (**a**).

To evaluate the efficiency of MWCNTs as nucleating agents, it is necessary to compare their effect with that of PCL self-nuclei. Self-nucleation is a thermal protocol for the production of self-nuclei within a polymer melt, such that the nucleation density can be greatly increased. In theory, the best nucleating agent for any polymer is its own crystal fragments or chain segments with residual crystal memory [25,26,27]. Figure 14 shows the experimental data obtained during an SN experiment for neat PCL. The cooling scans after the isothermal step at *T_s_* are presented in Figure 14a, and the subsequent heating scans are shown in Figure 14b. The dashed line indicates the PCL crystallization and melting temperatures under standard conditions. The three SN domains are described below as defined by Fillon et al. [25].

*Domain I (The Melting Domain).* The polymer is under *Domain I* when complete melting occurs and the crystalline history of the material is erased. All crystalline memory is erased and the melt is isotropic. For the PCL studied in Figure 14, *Domain I* is found at *T_s_* larger or equal to 61 °C, since no change was detected in *T_c_* when compared to the standard *T_c_*. Both the crystallization and melting DSC scans are identical within *Domain I*.

*Domain II (The Self-Nucleation Domain).* In this domain, the *T_s_* range employed is low enough to produce self-nuclei, but high enough to avoid annealing. Therefore, *Domain II* is easily identified after 5 min at a given *T_s_*, because the peak crystallization temperature of the sample increases compared the standard value. The start of *Domain II* for the PCL sample in Figure 14a occurred at a *T_s_* = 60 °C, since the sample was self-nucleated without any annealing. The minimum *T_s_* within *Domain II* is defined as the “ideal self-nucleation temperature” (*T_s,ideal_*), a temperature that should be accurately determined. This is the temperature that causes maximum self-nucleation (maximum increase in *T_c_*) without annealing. The subsequent melting curve in Figure 14b does not reveal any sign of annealing. In this domain, the nucleation density is enhanced, which makes the crystallization of PCL possible at higher temperatures.

*Domain III (The Self-Nucleation and Annealing Domain)*. When *T_s_* is too low, partial melting occurs and the unmolten crystals anneal during the 5 min at *T_s_*. Figure 14b shows that, when *T_s_* < 60 °C, the melting endotherm exhibits a small high temperature peak that is a result of the melting of annealed crystals. At this *T_s_*, the crystallization exotherm shows a high-temperature tail which reveals that the sample is in *Domain III*.

**Figure 15:**
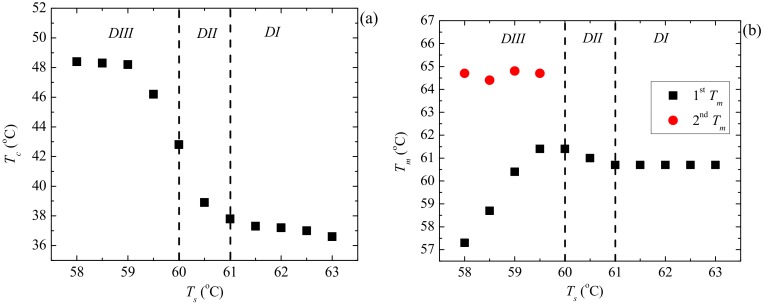
Dependence of (**a**) crystallization and (**b**) melting peak temperatures of neat PCL on *T_s_*.

Figure 15 shows the location of the three self-nucleation domains for the PCL sample. The vertical dashed lines indicate the temperatures at which the material experiences a self-nucleation domain transition. The *T_c_*-values are constant in *Domain I* and increase as the *T_s_*-value crosses over to *Domain II*, as expected [25,26]. Since 60 °C is the lowest *T_s_*-value in *Domain II*, it is called the ideal self-nucleation temperature, because it is the temperature at which there is maximum self-nucleation without any annealing. Employing the ideal *T_s_* (60 °C), the *T_c_* corresponding to the ideal *T_s_* should be used as the maximum crystallization temperature (*T_c,max_*) when determining the nucleation efficiency of the nanofiller. For the PCL used in this study, *T_c,max_* is 42.8 °C.

**Figure 16:**
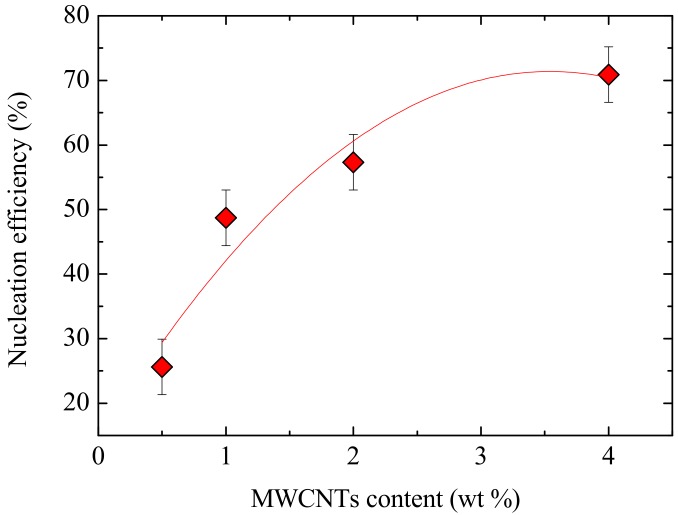
Nucleation efficiency as a function of MWCNT content. The experimental points are fitted with a polynomial fit to guide the eye.

The efficiency of the MWCNTs as nucleating agents for the PCL matrix was calculated according to Equation (3), which was proposed by Fillon et al. [54].

(3) $$NE=\frac{T_{c,NA}-\;T_{c,PCL}}{T_{c,max}-\;T_{c,PCL}}\times 100$$

where *T_c,NA_* is the peak *T_c_*-value determined from non-isothermal DSC cooling run for a sample of the polymer with the nucleating agent (NA), *T_c,PCL_* is the peak *T_c_*-value for neat PCL after its crystalline history has been erased (31.2 °C), (also determined from non-isothermal DSC cooling scan) and *T_c,max_* is the maximum peak crystallization temperature determined after neat PCL has been self-nucleated at the ideal *T_s_* (42.8 °C) [27,54]. Figure 16 shows the percentage nucleation efficiency of MWCNTs in the PCL/(PC/MWCNT) nanocomposites.

The nucleating efficiency increases with increasing MWCNT content. Notice that, if the blends were totally immiscible, the MWCNTs would be trapped inside the PC phase and the increase in MWCNTs would probably cause no increase in nucleation efficiency. In this case, the nucleation efficiency clearly increases with MWCNTs, a result which is also consistent with the partial miscibility of the blends. The fast increase in the nucleation efficiency is observed until a concentration of 1 wt % is reached, which is in line with the percolation threshold obtained in the previous section as well as the DSC results. At MWCNT concentrations above 1 wt %, the increase is slow, which corresponds to a saturation effect due to an agglomeration of MWCNTs and PC crystallization.

The increase in nucleation efficiency (even if highly significant, up to 70%) is less than expected, as literature results indicate that MWCNTs can super nucleate PCL (i.e., can produce nucleation efficiencies larger than 100% with loading as low as 1% or less) [16,19]. In our case, the nucleating efficiency is lower due to limited phase mixing between the PC-rich and the PCL-rich phases. Only a limited quantity of MWCNTs can penetrate the PCL-rich phase and therefore contribute in nucleating PCL, while most of the MWCNTs agglomerated in the PC-rich phase.

### 3.6. Overall Isothermal Crystallization Studied by DSC

**Figure 17:**
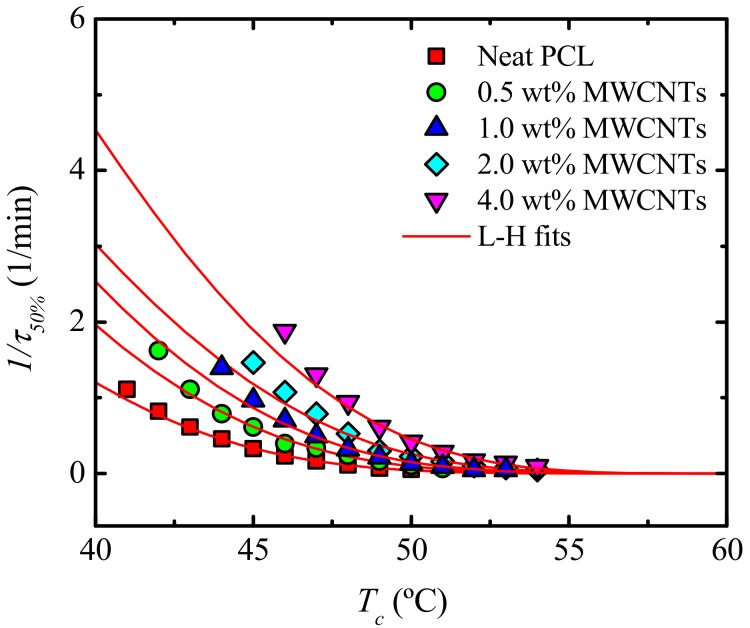
Overall crystallization rate (1/*τ*_50%_) as a function of isothermal crystallization temperature (*T_c_*) for neat PCL and for the PCL/(PC/MWCNT) nanocomposites. The red solid lines represent fits to the LH theory.

The influence of the MWCNTs as well as PC (both components of the masterbatch employed) at different contents, over the isothermal crystallization kinetics of the PCL are studied. Figure 17 shows the inverse of the half crystallization time (1/*τ*_50%*Exp*_), which is proportional to the overall crystallization rate as a function of isothermal crystallization temperatures (*T_c_*) for neat PCL and the nanocomposites. The *T_c_* range for neat PCL is lower than that for the nanocomposites. This indicates that a larger degree of supercooling is needed for neat PCL to crystallize, while the nanocomposites crystallize more easily than does neat PCL, because of the nucleation effect that they have on the PCL-rich phase.

**Figure 18:**
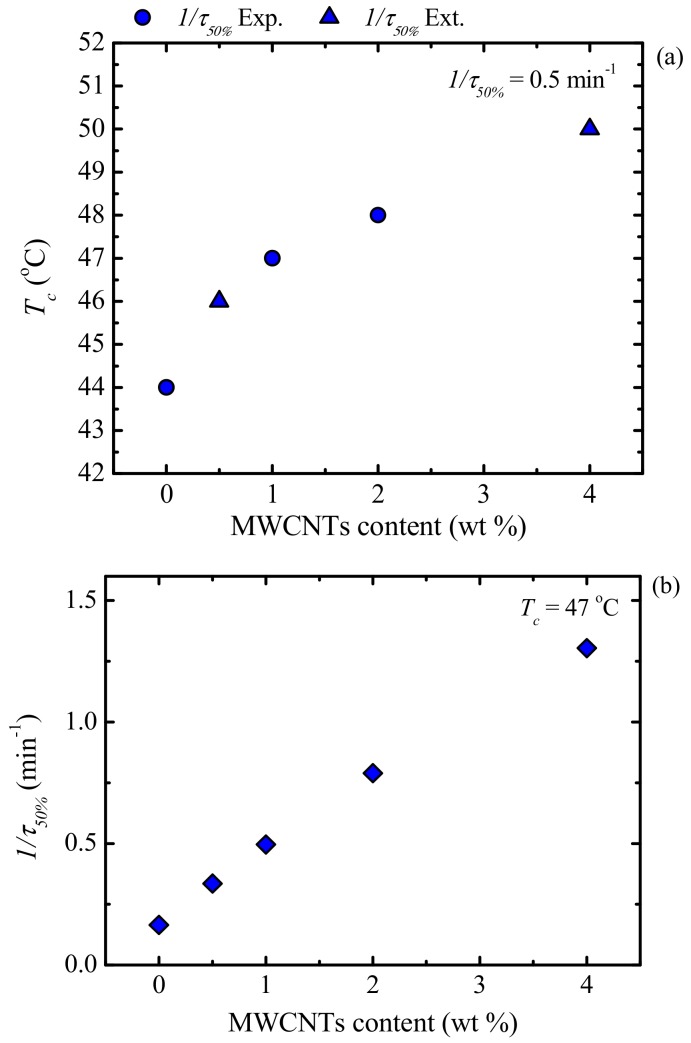
(**a**) Crystallization temperature as a function of MWCNT content at constant 1/*τ*_50%_ = 0.5 min^−1^; (**b**) overall crystallization rate as a function of MWCNT content at constant *T_c_* = 47 °C.

Another way to examine the results presented in Figure 17, is by taking the values of the crystallization temperature for which the blends reach a constant value of 1/*τ*_50%_ (i.e., 0.5 min^−1^) (Figure 18a) and the 1/*τ*_50%_-values at a constant *T_c_* (i.e., 47 °C) (Figure 18b), as a function of MWCNT content. Figure 18a shows the experimental and extrapolated data using the Lauritzen and Hoffman (L-H) theory, which is explained in detail in Section 3.6.2. It is clear that nucleation produces an interesting practical effect, as a higher *T_c_*-value is needed to reach the same overall crystallization rate with increasing MWCNT content in the PCL-rich phase. This result is in agreement with previous works [16,17,19], where an increase in MWCNT content resulted in an increase in the peak crystallization temperature of PCL during non-isothermal crystallization.

**Figure 19:**
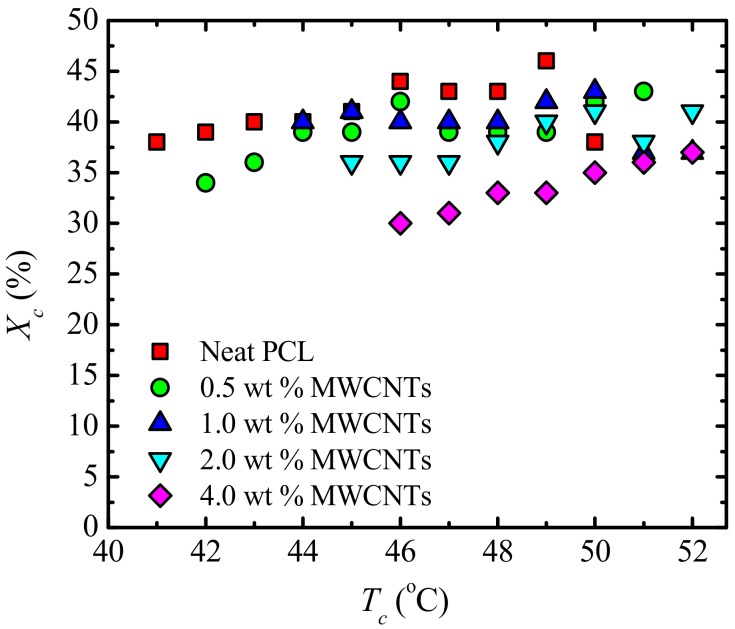
Relative crystallinity (*X_c_*) as a function of isothermal crystallization temperature (*T_c_*) for neat PCL and the PCL/(PC/MWCNT) nanocomposites.

In Figure 18b, the overall crystallization rate of the PCL-rich phase at a constant crystallization temperature increases with MWCNT loading, due to the nucleating effect of the MWCNTs. Despite the nucleating effect of MWCNTs, a reduction in the percentage crystallinity (*X_c_*) of PCL is observed (see Figure 19), especially at high PC concentrations. It must be remembered (see Table 1) that, since the nanocomposites are prepared by mixing PCL and a masterbatch, as the MWCNT content increases, so does the PC fraction in the blends. This decrease in *X_c_* of the PCL matrix (Figure 19), during isothermal crystallization, is due to the presence of the PC-rich phase, which is able to crystallize because of the plasticization effect of the PCL component.

#### 3.6.1. Fitting DSC Isothermal Data to the Avrami Model

**Figure 20:**
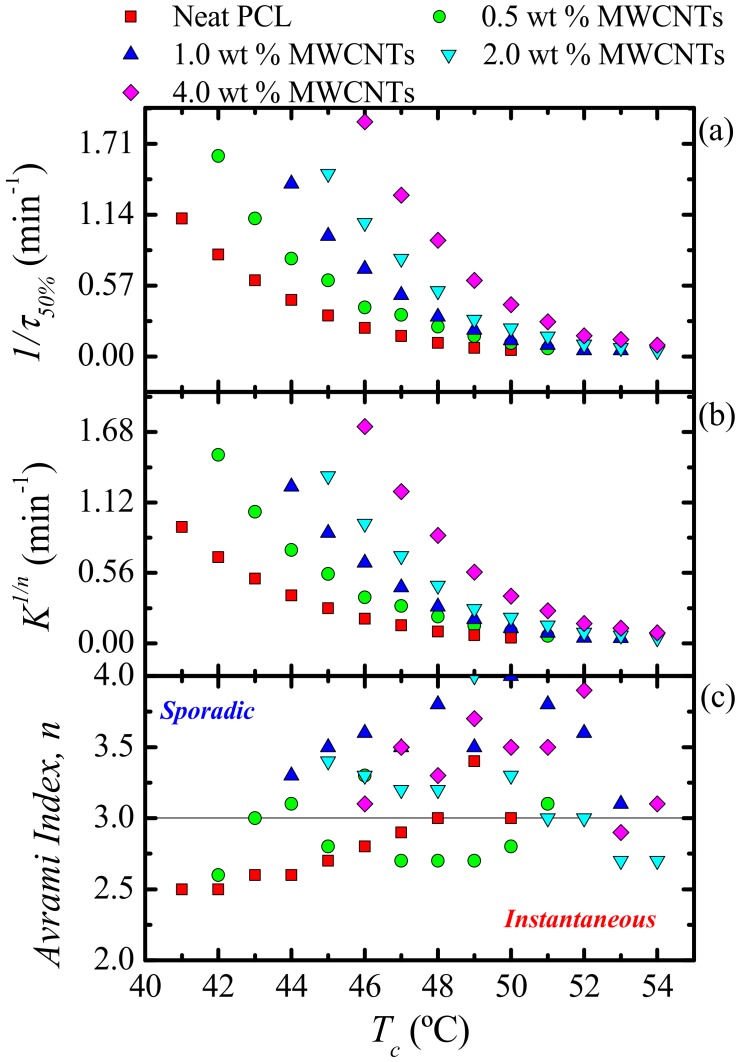
(**a**) Inverse of half crystallization times (1/*τ*_50%_) (**b**) Normalized crystallization constant of the Avrami model (*K*^1/*n*^) and (**c**) Avrami index (*n*) as a function of the isothermal crystallization temperature (*T_c_*) for all the samples.

The data obtained during the isothermal crystallization experiments were analyzed employing the Avrami equation, which can be expressed as follows [55]:

(4) $$1-V_{c}\left(t-t_{0}\right)=\exp(-K{\left(t-t_{0}\right)}^{n})$$

where t is the experimental time, $t_{0}$ is the induction time, $V_{c}$ is the relative volumetric transformed fraction, n is the Avrami index, and K is the overall crystallization rate constant. The procedure used to perform the fittings to the data was developed by Lorenzo et al. [29]. The kinetic parameters for all the investigated samples are plotted in Figure 20 and tabulated in Table S1.

Figure 20a shows 1/*τ*_50%_-values as a function of *T_c_*, whose trend was explained earlier in the discussion. The same trend is obtained with the *K*^1/*n*^-values of the Avrami model (see Figure 20b), since this constant is related to the overall crystallization kinetics as well. Figure 20c shows the *n*-values for all the samples, which depend on the dimensionality of the crystalline superstructure and on their nucleation kinetics [29,55].

The values of *n* for neat PCL are approximately 3 in the investigated *T_c_* range, which is an expected result for PCL [16,17]. A value of 3 indicates a spherulitic morphology with instantaneous nucleation, which is commonly observed in PCL homopolymers. Therefore, upon the addition of a nucleating agent, one would expect that the Avrami index would remain around 3 or would decrease (as the dimensionality of growth can switch from 3D to 2D when the nucleation density is greatly enhanced). Higher values than 3 for these PCL nanocomposites are not expected, especially when it has been demonstrated that MWCNTs are effective in nucleating PCL. Further studies are needed in order to understand the explanation of such unexpected results. Elsewhere in the literature [16,17,19], the authors reported decreasing *n*-values for the nanocomposites as compared to neat PCL.

#### 3.6.2. Overall Isothermal Crystallization Data Analyzed by the Lauritzen-Hoffman Model

The overall crystallization kinetics is determined by contributions of primary nucleation and growth. The Lauritzen-Hoffman (LH) nucleation and growth theory can be applied to the isothermal crystallization kinetics data collected from DSC. Even though the LH theory has received much criticism lately [56], it is still one of a few models that provide easy to use analytical expressions capable of fitting the experimental data over a wide supercooling range [57]. Figure 17 shows solid lines that represent the mathematical fit of LH theory, which can be applied to the DSC overall crystallization data according to Equation (5).

(5) $$\frac{1}{\tau_{50\%}}\left(T\right)=\;\frac{1}{\tau_{0}}\exp\left(\frac{-U^{*}}{R(T_{c}-\;T_{\infty )}}\right)\exp\left(\frac{-K_{g}^{\tau }}{T_{c}\Delta T_{f}}\right)$$

where 1/*τ*_50%_ is the inverse of the experimental half-crystallization time, 1/*τ*_0_ a pre-exponential factor that includes the nucleation and growth, U* the activation energy for the transport of the chains to the growing front (a value of 1500 cal mol^−1^ is usually employed), *R* the gas constant, *T_c_* the isothermal crystallization temperature (K), and *T_∞_* the temperature at which chain mobility ceases (usually taken as *T_g_* − 30 K). Δ*T* = supercooling ($T_{m}^{o}-\;T_{c})$, and *K_g_^τ^* is a constant related to the energy barrier for crystallization and growth.

According to Figure 17, the lines can adequately fit the overall crystallization rate as a function of *T_c_* for the explored range. The fittings were useful in constructing Figure 20, which was produced by extrapolating unavailable data in specific temperature ranges. Additionally, it was found, as expected and reported before in similar studies [16,17,19], that *K_g_^τ^*-values (proportional to the energy barrier for overall crystallization) decrease when the nanotube content increases as a result of their nucleating ability.

### 3.7. Thermal Conductivity

**Figure 21:**
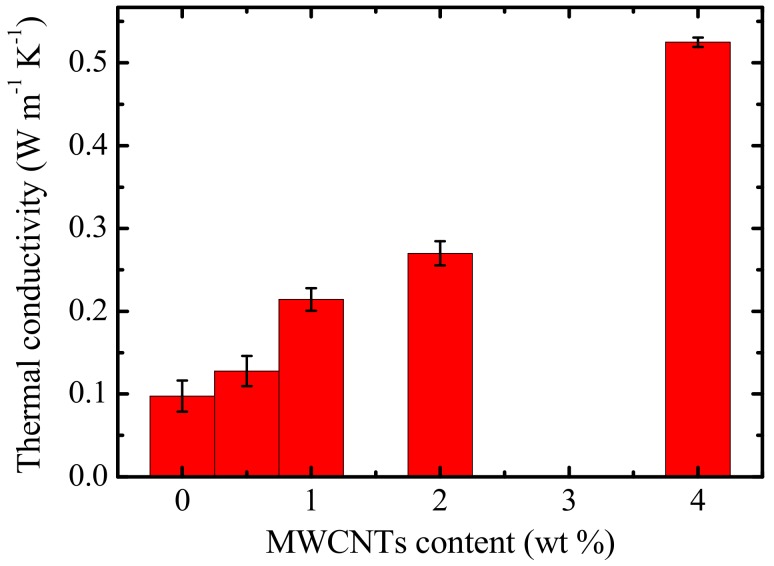
Influence of MWCNT content on the thermal conductivities of the nanocomposites.

The thermal conductivities of neat PCL and its PC/MWCNTs containing nanocomposites are shown in Figure 21. It is well known that phonon transport is the main mechanism for heat conduction in conductive polymer samples. Phonons transfer heat energy through interactions with each other and with subatomic particles [6,7,8]. The thermal conductivity values of the nanocomposites increased as MWCNT content increased. Since the thermal conductivity of carbon nanotubes ranges between 650 and 10,000 W m K^−1^, and the thermal conductivity of a typical polymer ranges between 0.1 and 0.3 W m K^−1^ [10], the improvement in the thermal conductivity is most probably caused by the increasing numbers of high thermal conductivity MWCNTs in the blend composites. Since the MWCNTs were fairly well dispersed in the PCL/PC blend (although there were pockets where the MWCNTs are more concentrated, see already discussed SEM and TEM results), the MWCNTs were positioned closer to each other as the MWCNT content increased, which gave rise to more effective transport of the phonons through the nanocomposite, which improved the transportation of heat by high frequency phonon vibration, leading to higher thermal conductivities. In this case, the thermal conductivity does not seem to be affected by the aggregation of carbon nanotubes, which, as discussed previously, was detected when the filler level increased to 4%.

### 3.8. Tensile Properties

The mechanical properties of PCL and the nanocomposites were investigated employing tensile testing. These properties depend upon the interfacial interaction between the nanofiller and the different components in the polymer blend, chain stiffness, and the crystallinities of the individual components in the blend. This implies that, to utilize the reinforcing capability of carbon nanotubes and to maximize the mechanical properties of the nanocomposites, strong interfacial bonding is necessary. The extent of interaction depends on how well the filler is dispersed in the matrix [58].

**Figure 22:**
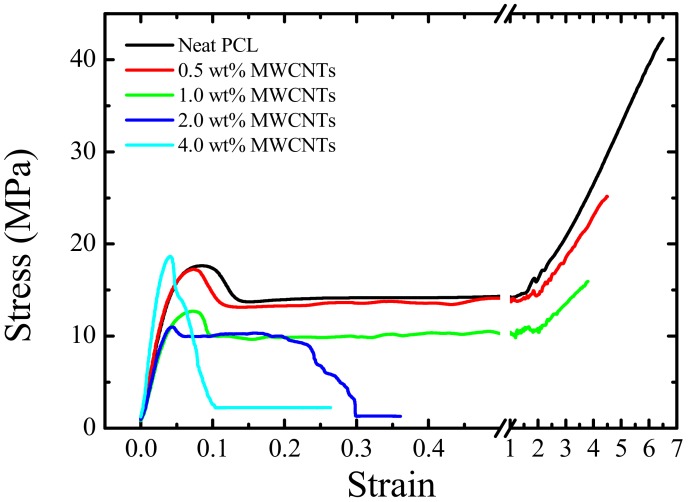
Stress–strain curves for neat PCL and the nanocomposites.

Considering that the nanocomposites prepared here involve the increasing addition of both PC and MWCNTs to PCL, it is expected that the ductility of the PCL significantly decreases while the modulus increases. Table 3 presents the tensile tests results, which partially corroborate the expected trends. The strain at break significantly decreases as the masterbatch content increases (see Figure 22), a trend proportional to a reduction in ductility. Additionally, the elastic modulus is not significantly affected, until the maximum amount of masterbatch was used. In this last case, the value increased from 388 MPa for neat PCL to 592 MPa for the nanocomposite.

PC is a more rigid polymer than PCL. In the present blends, PC addition caused a small increase in the *T_g_* of the PCL phase due to the partial miscibility. Additionally, the presence of PC-rich inclusions in a PCL matrix can act as stress concentrators that may trigger earlier fracture nucleation and propagation.

On the other hand, MWCNTs are known to increase overall rigidity of the polymer matrix to which they are added, when the dispersion is adequate and when there are strong interactions with the polymer matrix. In this case, the interactions may not be very strong with the PCL matrix, and the fact that the density of MWCNTs is higher in the PC-rich phase than in the PCL-rich phase probably has a stronger stress concentration effect and is less effective at enhancing the elastic modulus of the nanocomposites. This may be the reason why the positive effect on the elastic modulus can only be obtained at large masterbatch loadings. As both PC and MWCNT additions induce enhanced rigidity and stress concentrations in the PCL matrix, the stress at break is also seen to decrease with masterbatch additions.

Although increases in tensile strength, similar to that of the Young’s modulus, have been reported in CNT-filled nanocomposites, the local nature of the shear yielding process, usually leads to constant or even decreasing values as CNT content increases [59]. In this case, the yield stress of PCL first decreases slightly with masterbatch additions and then recovers at the maximum concentration of MWCNTs.

## 4. Conclusions

In light of an analysis of the DSC, SEM, DMA, TEM, and AFM results, it can be concluded that the PC and PCL blends prepared in this work are partially miscible. Two phases were formed: (1) A PC-rich phase, where a small amount of PCL chains are present and can plasticize the PC component, such that it can crystallize, and (2) a PCL-rich phase, where the amount of PC chains present is very small, such that changes in the *T_g_* of the PCL phase are much smaller than those predicted by the Fox equation. Due to partial miscibility and the establishment of PC-rich and PCL-rich phases, a fair number of MWCNTs diffused from the PC-rich phase to the PCL-rich phase as evidenced by the SEM and AFM images, but their diffusion depends on the PC (and MWCNT content) in the blend.

Standard DSC measurements demonstrated an increase in *T_c_* with increasing MWCNT content due to the nucleation effect caused by the presence of MWCNTs that penetrated into the PCL-rich phase (as was demonstrated morphologically by SEM and AFM images). The nucleating efficiency is, however, low, due to limited phase mixing between the PC-rich and the PCL-rich phases. Only a limited quantity of MWCNTs can penetrate the PCL-rich phase and therefore contribute in nucleating PCL, while most of the MWCNTs agglomerated in the PC-rich phase at high MWCNT loadings (e.g., 4%). The nucleation effects saturated at a 1 wt % MWCNT concentration on the PCL-rich phase. This was corroborated by the determination of the dielectric percolation threshold, which ranged between 0.5 and 1.0 wt % MWCNTs.

Isothermal crystallization experiments performed by DSC showed an increase in the overall crystallization kinetics of PCL with increases in MWCNTs as a result of their nucleating effect. Despite the nucleating effect of MWCNTs, a reduction in the percentage crystallinity of PCL was observed especially at high PC concentrations. This was attributed to the presence of the PC-rich phase, which was able to crystallize in view of the plasticization effect of the PCL component.

The thermal conductivities and tensile properties of the nanocomposites were generally enhanced with the addition of MWCNTs.

## Figures and Tables

**Table 1:** Weight percentages of the components in the nanocomposites.

PCL (%)	PC (%)	MWCNTs (%)
100	0	0
97	2.55	0.45
93	5.95	1.05
87	11.05	1.95
73	22.95	4.05

**Table 2:** Calculated values of *d*-spacing (from WAXS (Wide Angle X-ray Scattering) experiments) and long period (d*, obtained from SAXS (Small Angle X-ray Scattering) experiments) for the neat PCL and its nanocomposites.

Sample	*d*-Spacing (nm)/(plane)	d * (nm)
Neat PCL	0.378 (110)	19.8
	0.343 (200)	
	0.400 (111)	
93/(6/1) *w*/*w* PCL/(PC/MWCNTs)	0.378 (110)	19.9
	0.344 (200)	
	0.400(111)	
73/(23/4) *w*/*w* PCL/(PC/MWCNTs)	0.464 *	17.2 **
	0.378 (110)	
	0.344 (200)	
	0.400 (111)	

* PC signal; ** overlap of PC and PCL signals.

**Table 3:** Summary of tensile testing results for neat PCL and the nanocomposites.

*w*/*w* PCL/(PC/MWCNTs)	$\sigma_{b}$/MPa	$\varepsilon_{b}$/%	E/MPa	$\sigma_{y}$/MPa
100/0	34.3 ± 12.9	578 ± 151	388 ± 29	16.2 ± 1.9
97/(2.5/0.5)	15.9 ± 7.1	285 ± 152	354 ± 77	13.7 ± 3.3
93/(6/1)	10.3 ± 5.0	154 ± 198	352 ± 28	13.6 ± 1.0
87/(11/2)	6.9 ± 1.9	22.8 ± 2.0	336 ± 33	11.3 ± 4.1
73/(23/4)	15.6 ± 0.9	4.3 ± 0.7	592 ± 62	17.1 ± 1.4

$\sigma_{b}\;—$stress at break; $\varepsilon_{b}$—strain at break; E—Young’s modulus; $\sigma_{y}$—yield strength.

## Supplementary Materials

The following are available online at www.mdpi.com/2073-4360/9/12/709/s1. Figure S1: Heating of PCL after isothermal crystallization at 41 °C, Table S1: Kinetic parameters for all the investigated samples during isothermal crystallization.

## References

[1] Vroman I., Tighzert L. (2009). Biodegradable polymers. Materials.

[2] Morent R., De Geyter N., Desmet T., Dubruel P., Leys C. (2011). Plasma surface modification of biodegradable polymers: A Review. Plasma Process. Polym..

[3] Reddy M.M., Vivekanandhan S., Misra M., Bhatia S.K., Mohanty A.K. (2013). Biobased plastics and bionanocomposites: Current status and future opportunities. Prog. Polym. Sci..

[4] Babu R.P., Connor K.O., Seeram R. (2013). Current progress on bio-based polymers and their future trends. Prog. Biomater..

[5] George J.J., Bhadra S., Bhowmick A.K. (2010). Influence of carbon-based nanofillers on the electrical and dielectric properties of ethylene vinyl acetate nanocomposites. Polym. Compos..

[6] Wurm A., Lellinger D., Minakov A.A., Skipa T., Pötschke P., Nicula R., Alig I., Schick C. (2014). Crystallization of poly(ε-caprolactone)/MWCNT composites: A combined SAXS/WAXS, electrical and thermal conductivity study. Polymer.

[7] Chin S.J., Vempati S., Dawson P., Knite M., Linarts A., Ozols K., McNally T. (2015). Electrical conduction and rheological behaviour of composites of poly(ε-caprolactone) and MWCNTs. Polymer.

[8] Pötschke P., Villmow T., Krause B. (2013). Melt mixed PCL/MWCNT composites prepared at different rotation speeds: Characterization of rheological, thermal, and electrical properties, molecular weight, MWCNT macrodispersion, and MWCNT length distribution. Polymer.

[9] Ma P.C., Siddiqui N.A., Marom G., Kim J.K. (2010). Dispersion and functionalization of carbon nanotubes for polymer-based nanocomposites: A review. Compos. A Appl. Sci. Manuf..

[10] Bhattacharya M. (2016). Review: Polymer nanocomposites—A comparison between carbon nanotubes, graphene, and clay as nanofillers. Materials.

[11] Prashantha K., Soulestin J., Lacrampe M.F., Krawczak P., Dupin G., Claes M. (2009). Masterbatch-based multi-walled carbon nanotube filled polypropylene nanocomposites: Assessment of rheological and mechanical properties. Compos. Sci. Technol..

[12] Jajam K.C., Rahman M.M., Hosur M.V., Tippur H.V. (2014). Fracture behavior of epoxy nanocomposites modified with polyol diluent and amino-functionalized multi-walled carbon nanotubes: A loading rate study. Compos. A Appl. Sci. Manuf..

[13] Rahman M.M., Hosur M., Zainuddin S., Jajam K.C., Tippur H.V., Jeelani S. (2012). Mechanical characterization of epoxy composites modified with reactive polyol diluent and randomly-oriented amino-functionalized MWCNTs. Polym. Test..

[14] Wu T., Chen E., Lin Y., Chiang M., Chang G. (2008). Preparation and characterization of melt-processed polycarbonate/multiwalled carbon nanotube composites. Polym. Eng. Sci..

[15] Robeson L.M. (2007). Polymer Blends: A Comprehensive Review.

[16] Trujillo M., Arnal M.L., Müller A.J., Dubois P. (2012). Supernucleation and crystallization regime change provoked by MWCNT addition to poly(ε-caprolactone). Polymer.

[17] Pérez R.A., López J.V., Hoskins J.N., Zhang B., Grayson S.M., Casas M.T., Puiggalí J., Müller A.J. (2014). Nucleation and antinucleation effects of functionalized carbon nanotubes on cyclic and linear poly(ε-caprolactones). Macromolecules.

[18] Yeh J.T., Yang M.C., Wu C.J., Wu C.S. (2009). Preparation and characterization of biodegradable polycaprolactone/multiwalled carbon nanotubes nanocomposites. J. Appl. Polym. Sci..

[19] Vega J.F., Fernández-Alcázar J., Lόpez J.V., Michell R.M., Pérez-Camargo R.A., Ruelle B., Martίnez-Salazar J., Arnal M.L., Dubois P., Müller A.J. (2017). Competition between supernucleation and plasticization in the crystallization and rheological behavior of PCL/CNT-based nanocomposites and nanohybrids. J. Polym. Sci. Part B Polym. Phys..

[20] Maiti S., Suin S., Shrivastava N.K., Khatua B.B. (2014). Low percolation threshold and high electrical conductivity in melt-blended polycarbonate/multiwall carbon nanotube nanocomposites in the presence of poly(ɛ-caprolactone). Polym. Eng. Sci..

[21] Qiu Z., Wang H., Xu C. (2011). Crystallization, mechanical properties, and controlled enzymatic degradation of biodegradable poly(ε-caprolactone)/multi-walled carbon nanotubes nanocomposites. J. Nanosci. Nanotechnol..

[22] Lee H.H., Shin U.S., Jin G.Z., Kim H.W. (2011). Highly homogeneous carbon nanotube polycaprolactone composites with various and controllable concentrations of ionically-modified MWCNTs. Bull. Korean Chem. Soc..

[23] Pan L., Pei X., He R., Wan Q., Wang J. (2012). Multiwall carbon nanotubes/polycaprolactone composites for bone tissue engineering application. Colloids Surf. B.

[24] Kasaliwal G.R., Göldel A., Pötschke P., Heinrich G. (2011). Influences of polymer matrix melt viscosity and molecular weight on MWCNT agglomerate dispersion. Polymer.

[25] Fillon B., Wittmann J.C., Lotz B., Thierry A. (1993). Self-nucleation and recrystallization of isotactic polypropylene (α phase) investigated by differential scanning calorimetry. J. Polym. Sci. Part B Polym. Phys..

[26] Lorenzo A.T., Arnal M.A., Sánchez J.J., Müller A.J. (2006). Effect of annealing time on the self-nucleation behaviour of semicrystalline polymers. J. Polym. Sci. Part B Polym. Phys..

[27] Müller A.J., Arnal M.L. (2005). Thermal fractionation of polymers. Prog. Polym. Sci..

[28] Michell R.M., Mugica A., Zubitur M., Müller A.J. (2017). Self-nucleation of crystalline phases within homopolymers, polymer blends, copolymers, and nanocomposites. Adv. Polym. Sci..

[29] Lorenzo A.T., Arnal M.L., Albuerne J., Müller A.J. (2007). DSC isothermal polymer crystallization kinetics measurements and the use of the Avrami equation to fit the data: Guidelines to avoid common problems. Polym. Test..

[30] Hoffman J.D., Weeks J.J. (1962). Melting process and the equilibrium melting temperature of polychlorotrifluoroethylene. J. Res. Natl. Bur. Stand. Sect. A.

[31] Menczel J.D., Prime R.B. (2009). Thermal Analysis of Polymers: Fundamentals and Applications.

[32] Zhang Y., Han J.H. (2010). Crystallization of high-amylose starch by the addition of plasticizers at low and intermediate concentrations. J. Food Sci..

[33] Müller A.J., Paredes E. (1985). Melting behavior, mechanical properties and fracture of crystallized polycarbonates. Lat. Am. J. Metall. Mater..

[34] Balsamo V., Calzadilla N., Mora G., Müller A.J. (2001). Thermal characterization of polycarbonate/polycaprolactone blends. J. Polym. Sci. Part B Polym. Phys..

[35] Hu H., Dorset D.L. (1990). Crystal structure of poly(iɛ-caprolactone). Macromolecules.

[36] Pötschke P., Bhattacharyya A.R., Janke A., Goering H. (2003). Melt mixing of polycarbonate/multi-wall carbon nanotube composites. Compos. Interface.

[37] Guo J., Liu Y., Prada-Silvy R., Tan Y., Azad S., Krause B., Pötschke P., Grady B.P. (2014). Aspect ratio effects of multi-walled carbon nanotubes on electrical, mechanical, and thermal properties of polycarbonate/MWCNT composites. J. Polym. Sci. Part B Polym. Phys..

[38] Castillo F.Y., Socher R., Krause B., Headrick R., Grady B.P., Prada-Silvy R., Pötschke P. (2011). Electrical, mechanical, and glass transition behavior of polycarbonate-based nanocomposites with different multi-walled carbon nanotubes. Polymer.

[39] Cruz C.A., Paul D.R., Barlow J.W. (1979). Polyester-polycarbonate blends. IV. Poly(ε-caprolactone). J. Appl. Polym. Sci..

[40] Chun Y.S., Park J., Sun J.B., Kim W.N. (2000). Blends of polycarbonate and poly(ε-caprolactone) and the determination of the polymer-polymer interaction parameter of the two polymers. J. Polym. Sci. Part B Polym. Phys..

[41] Ketelaars A.A.J., Papantoniou Y., Nakayama K. (1997). Analysis of the density and the enthalpy of poly(ε-caprolactone)-polycarbonate blends: Amorphous phase compatibility and the effect of secondary crystallization. J. Appl. Polym. Sci..

[42] Jonza J.M., Porter R.S. (1986). Bisphenol A polycarbonate/poly(ε-caprolactone) blends: Melting point depression and reactivity. Macromolecules.

[43] Herrera D., Zamora J.C., Bello A., Grimau M., Laredo E., Müller A.J., Lodge T.P. (2005). Miscibility and crystallization in polycarbonate/poly(ε-caprolactone) blends: Application of the self-concentration model. Macromolecules.

[44] Cheung Y.W., Stein R.S., Chu B., Wu G. (1994). Evolution of crystalline structures of poly(ε-caprolactone)/polycarbonate blends. 1. Isothermal crystallization kinetics as probed by synchrotron small-angle X-ray scattering. Macromolecules.

[45] Young R.J., Lovell P.A. (2011). Introduction to Polymers.

[46] Favis B.D., Therrien D. (1991). Factors influencing structure formation and phase size in an immiscible polymer blend of polycarbonate and polypropylene prepared by twin-screw extrusion. Polymer.

[47] Shih K.S., Beatty C.L. (1987). Blends of polycarbonate and poly(hexamethylene sebacate): II. Effect of molecular weight on compatibility. Polym. Eng. Sci..

[48] Oyarzabal A., Cristiano-Tassi A., Laredo E., Newman D., Bello A., Etxeberría A., Eguiazabal J.I., Zubitur M., Mugica A., Müller A.J. (2017). Dielectric, mechanical and transport properties of bisphenol A polycarbonate/graphene nanocomposites prepared by melt blending. J. Appl. Polym. Sci..

[49] Greenhoe B.M., Hassan M.K., Wiggins J.S., Mauritz K.A. (2016). Universal power law behavior of the AC conductivity versus frequency of agglomerate morphologies in conductive carbon nanotube reinforced epoxy networks. J. Polym. Sci. Part B Polym. Phys..

[50] Belaabed B., Lamouri S., Naar N., Bourson P., Hamady S.O.S. (2010). Polyaniline-doped benzene sulfonic acid/epoxy resin composites: Structural, morphological, thermal and dielectric behaviors. Polym. J..

[51] Arup C. (2009). Polyaniline/silver nanocomposites. Dielectric properties and ethanol vapor sensitivity. Sens. Actuators B.

[52] Saeed K., Park S.Y. (2007). Preparation and properties of multiwalled carbon nanotube/polycaprolactone nanocomposites. J. Appl. Polym. Sci..

[53] Pötschke P., Abdel-Goad M., Alig I., Dudkin S., Lellinger D. (2004). Rheological and dielectrical characterization of melt mixed polycarbonate-multiwalled carbon nanotube composites. Polymer.

[54] Fillon B., Lotz B., Thierry A., Wittmann J.C. (1993). Self-nucleation and enhanced nucleation of polymers. Definition of a convenient calorimetric “efficiency scale” and evaluation of nucleating additives in isotactic polypropylene (α phase). J. Polym. Sci. Part B Polym. Phys..

[55] Avrami M. (1941). Granulation, phase change, and microstructure kinetics of phase change III. J. Chem. Phys..

[56] Reiter G., Strobl G.R. (2007). Progress in Understanding Polymer Crystallization.

[57] Lorenzo A.T., Müller A.J. (2008). Estimation of the nucleation and crystal growth contributions to the overall crystallization energy. J. Polym. Sci. Part B Polym. Phys..

[58] Poveda R.L., Gupta N. (2016). Carbon Nanofiber Reinforced Polymer Composites.

[59] Meng H., Gui G.X., Fang P.F., Yang R. (2008). Effects of acid- and diamine-modified MWNTs on the mechanical properties and crystallization behavior of polyamide 6. Polymer.

