# Supplementary material for: Morphology, Nucleation, and Isothermal Crystallization Kinetics of Poly(ε-caprolactone) Mixed with a Polycarbonate/MWCNTs Masterbatch
Source: Polymers (Basel). 2017 Dec 13;9(12):709. doi: 10.3390/polym9120709 (PMC6418913; doi:10.3390/polym9120709)
Supplement: Supplementary file 1 [file polymers-09-00709-s001.pdf]

Supplementary Materials

Morphology, nucleation, and isothermal crystallization kinetics of poly(ε-caprolactone) mixed with a polycarbonate/MWCNTs masterbatch

Thandi P. Gumede, Adriaan S. Luyt, Mohammad K. Hassan, Ricardo A. Pérez-Camargo, Agnieszka Tercjak, Alejandro J. Müller

Simultaneous SAXS/WAXS experiments

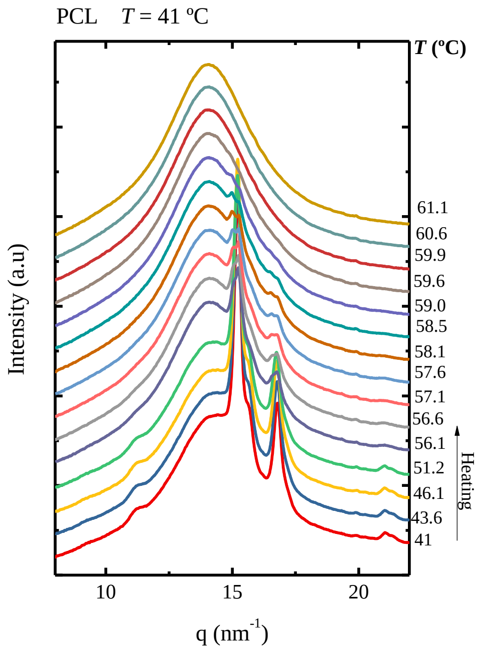

Figure S1. Heating of PCL after isothermal crystallization at 41 ºC.

Fitting of DSC isothermal data to the Avrami model

The kinetic parameters for all the investigated samples are shown in Table S1. It is worth to noting that a conversion range of approximately 3–20% was used and this corresponds to the primary crystallization range where the Avrami analysis is most adequate. In such a range the correlation coefficients of the fit are mostly in excess of 0.999 (Table S1). For all the samples studied, the half crystallization times for the experimental data ($\tau_{50\%\ Exp}$) and the Avrami fittings ($\tau_{50\%\ Theo}$) are almost the same, which indicates that the Avrami model predicts very well the crystallinity up to 50% relative crystallinity.

Table S1. Kinetic parameters for all the investigated samples during isothermal crystallization.

PCL/(PC/MWCNTs) Sample	T_c [°C]	t_0 [min]	ΔH [J.g^{-1}]	V_c range [%]	n	K [min^{-n}]	R^2	$\tau_{50\%\ Theo}$ [min]	$\tau_{50\%\ Exp}$ [min]	$(\tau_{50\%\ Exp})^{-1}$ [min^{-1}]	X_c [%]
100/0 w/w	41.0	0.35	52	3-20	2.5	8.21E-01	0.9998	0.93	0.90	1.1111	38
	42.0	0.52	53	3-20	2.5	3.86E-01	0.9999	1.26	1.22	0.8217	39
	43.0	0.50	54	3-20	2.6	1.77E-01	0.9999	1.68	1.63	0.6124	40
	44.0	0.67	55	3-20	2.6	8.13E-02	0.9999	2.25	2.20	0.4545	40
	45.0	1.03	56	3-20	2.7	3.20E-02	1.0000	3.09	3.03	0.3297	41
	46.0	1.13	60	3-20	2.8	1.03E-02	1.0000	4.40	4.37	0.2290	44
	47.0	1.63	59	3-20	2.9	3.64E-03	1.0000	6.16	6.07	0.1648	43
	48.0	1.82	59	3-20	3.0	8.24E-04	0.9999	9.15	9.03	0.1107	43
	49.0	1.02	63	3-20	3.4	8.36E-05	0.9995	14.76	14.43	0.0693	46
	50.0	2.27	51	3-20	3.0	7.94E-05	0.9976	20.21	18.87	0.0530	38
97/(2.5/05) w/w	42.0	0.35	45	3-20	2.6	2.86E+00	1.0000	0.58	0.62	1.6207	34
	43.0	0.47	48	3-20	3.0	1.14E+00	0.9998	0.85	0.90	1.1111	36

	44.0	0.43	52	3-20	3.1	3.95E-01	1.0000	1.20	1.27	0.7893	39
	45.0	0.67	52	3-20	2.8	1.89E-01	1.0000	1.59	1.63	0.6124	39
	46.0	0.67	55	3-20	3.3	3.62E-02	1.0000	2.44	2.53	0.3948	42
	47.0	1.46	51	3-20	2.7	3.79E-02	1.0000	2.97	2.98	0.3352	39
	48.0	2.25	51	3-20	2.7	1.50E-02	0.9999	4.22	4.18	0.2391	39
	49.0	3.10	52	3-20	2.7	5.04E-03	1.0000	6.15	6.10	0.1639	39
	50.0	4.63	56	3-20	2.8	1.39E-03	1.0000	9.35	9.40	0.1064	42
	51.0	5.67	57	3-20	3.1	1.54E-04	1.0000	15.45	15.58	0.0642	43
**93/(6/1) w/w**	44.0	0.32	50	3-20	3.3	2.07E+00	0.9998	0.72	0.72	1.3947	40
	45.0	0.50	52	3-20	3.5	6.30E-01	0.9998	1.03	1.03	0.9681	41
	46.0	0.53	50	3-20	3.6	2.02E-01	0.9999	1.42	1.42	0.7057	40
	47.0	0.77	50	3-20	3.5	5.81E-02	0.9999	2.01	2.02	0.4958	40
	48.0	0.95	51	3-20	3.8	9.17E-03	0.9999	3.11	3.13	0.3192	40
	49.0	1.48	53	3-20	3.5	3.20E-03	0.9995	4.71	4.65	0.2151	42
	50.0	1.92	55	3-20	4.0	2.19E-04	0.9999	7.65	7.72	0.1296	43

	51.0	3.68	47	3-20	3.8	7.89E-05	1.0000	10.77	10.85	0.0922	37
	52.0	2.53	47	3-20	3.6	1.23E-05	0.9959	21.82	20.35	0.0491	37
	53.0	14.85	30	3-20	3.1	5.35E-05	0.9997	20.34	19.70	0.0508	24
**87/(11/2) w/w**	45.0	0.33	43	3-20	3.4	2.63E+00	0.9999	0.68	0.68	1.4641	36
	46.0	0.57	43	3-20	3.3	8.52E-01	0.9999	0.94	0.93	1.0718	36
	47.0	0.67	43	3-20	3.2	3.10E-01	0.9998	1.29	1.27	0.7893	36
	48.0	0.90	45	3-20	3.2	8.34E-02	0.9998	1.92	1.90	0.5263	38
	49.0	0.80	47	3-20	4.1	4.93E-03	1.0000	3.30	3.35	0.2985	40
	50.0	1.98	48	3-20	3.3	5.26E-03	0.9999	4.39	4.40	0.2273	41
	51.0	3.75	45	3-20	3.0	2.87E-03	0.9999	6.26	6.20	0.1613	38
	52.0	5.35	49	3-20	3.0	6.52E-04	0.9999	10.34	10.22	0.0979	41
	53.0	11.22	46	3-20	2.7	5.95E-04	1.0000	14.13	14.08	0.0710	39
	54.0	20.92	44	3-20	2.7	1.96E-04	0.9996	20.38	21.63	0.0462	37
**73/(23/4) w/w**	46.0	0.28	30	3-20%	3.1	5.40E+00	1.0000	0.52	0.53	1.8762	30
	47.0	0.43	31	3-20%	3.5	1.93E+00	1.0000	0.75	0.77	1.3038	31

	48.0	0.47	33	3-20%	3.3	6.03E-01	0.9999	1.04	1.07	0.9372	33
	49.0	0.55	33	3-20%	3.7	1.21E-01	0.9999	1.61	1.63	0.6124	33
	50.0	0.85	35	3-20%	3.5	3.27E-02	0.9996	2.42	2.40	0.4167	35
	51.0	1.52	36	3-20%	3.5	8.79E-03	1.0000	3.54	3.58	0.2791	36
	52.0	2.20	37	3-20%	3.9	7.41E-04	1.0000	5.81	5.97	0.1676	37
	53.0	5.25	29	3-20%	2.9	2.22E-03	0.9996	7.50	7.23	0.1383	29
	54.0	9.62	24	3-20%	3.1	4.73E-04	1.0000	10.86	10.77	0.0929	24
